# Meeting abstracts from the 11th edition of the European conference on Rare Diseases & Orphan Products (ECRD) 2022

**DOI:** 10.1186/s13023-023-02707-4

**Published:** 2023-06-02

## P1

### Developing a Patient-Reported Outcome Measures (PROMs) repository for use in Rare Diseases

#### Céline Desvignes-Gleizes^1,*^, Benoit Arnould^1,*^, Mar Mañú Pereira^2,3^, Mariangela Pellegrini^2,4,*^, Gavin Mc Donough^5,*^, Sonia Bothorel^1^, Ana Rath^5,*^

##### ^1^Mapi Research Trust / ICON: 27 rue de la Villette, Lyon, France. ^2^ERN-EuroBloodNet, Hôpital St Louis / Université Paris 7 Paris, France. ^3^Rare anemia disorders research line Cancer and Blood disorders in Pediatrics Research Group. Vall d'Hebron Institut de Recerca, Barcelona, Spain. ^4^Assistance Publique Hôpitaux de Paris, Hôpital Saint Louis, Paris, France. ^5^INSERM, US14 - Orphanet, Paris, France

*Corresponding author: Celine.Desvignes-Gleizes@mapi-trust.org; Benoit.Arnould@iconplc.com; mar.manu@vhir.org; mariangela.pellegrini@aphp.fr; gavin.mc-donough@inserm.fr; ana.rath@inserm.fr

*Orphanet Journal of Rare Diseases* 2023: P1

**Background:** The European Rare Disease Research Coordination and Support Action (ERICA) consortium aims at promoting and disseminating the adoption of standardized PROMs for rare diseases. The dedicated working team (the WP3 group) facilitates the creation of a PROMs repository for use in clinical practice, evaluation of care and clinical research.

**Material and methods:** First, among the 4.000 questionnaires described in the Mapi Research Trust PROMs database, PROQOLID, we have selected PROMs developed and validated for rare diseasesand PROMs measuring specific functional impacts (such as mobility, self-care or communication). Second, we conducted a survey among European Reference Networks and patient organisations to collect additional PROMs of interest. Third, we developed coding rules for PROMs, based on the International Classification of Functioning, Disability and Health (ICF) code. The resulting ICF-coded PROMs had to match the ICF-coded functional impacts of rare diseases, generated by semi-structured interviews with the Orphanet Disability Questionnaire.

**Results:** The search in PROQOLID identified 279 PROMs developed in rare diseases and 200 PROMs measuring functional impacts. The survey identified 31 additional PROMs. A preliminary coding was conducted on a convenient sample of 10 PROMs including generic (EQ- 5D, SF-36), disease specific (Myasthenia Gravis-Quality of Life (MG-QOL) and Myasthenia Gravis-Activity of Daily Living (MG-ADL)) and function specific (Health Assessment Questionnaire (HAQ), National Eye Institute Visual Function Questionnaire -25 (NEI-VFQ-25)) PROMs.

**Conclusion:** We have selected a first set of more than 500 PROMs eligible for inclusion in the repository. We have defined and tested PROMs coding rules, which will allow 600 rare diseases described through the Orphanet Disability Questionnaire to be matched with the relevant PROMs. The next steps are to implement the coding model to the full set of PROMs, to operationalize the repository platform for both researchers and clinicians and to routinely code new eligible PROMs.

## P2

### Let Us Talk: a communication skills training course for healthcare professionals working with Huntington’s disease

#### Filipa Júlio^1,2,*^, Astri Arnesen^1,3^, Beatrice De Schepper^1,4^, Dina De Sousa^1,5^, Danuta Lis^1,6^, Julia Oblitsova^1,9^, Svein Olaf Olsen^1,3^, Giorgos Papantoniou^1,7^, Saija Ristolainen-Kotimäki^1,8^, Marina Tretyakova^1,9^ and Zaynab Umakhanova^1,9^

##### ^1^European Huntington Association, Moerbeke (Waas), Belgium. ^2^Associação Portuguesa dos Doentes de Huntington, Lisboa, Portugal. ^3^Landsforeningen for Huntingtons Sykdom, Askøy, Norway. ^4^Huntington Liga, Moerbeke (Waas), Belgium. ^5^Scottish Huntington's Association, Paisley, Renfrewshire, UK. ^6^Polskie Stowarzyszenie Choroby Huntingtona, Warszawa, Poland. ^7^Huntington's Disease Association of Cyprus, Limassol, Cyprus. ^8^Suomen Huntington Yhdistys RY, Turku, Finland. ^9^Orphan People, Moscow, Russia

*Corresponding author: filipa@eurohuntington.org

*Orphanet Journal of Rare Diseases* 2023: P2

**Background:** After conducting a needs assessment in the Russian community affected by Huntington’s Disease (HD), the European Huntington Association (EHA) has identified significant challenges in the patients’ relationship with healthcare professionals. Accordingly, the EHA developed “Let Us Talk”, an experimental web-based communication skills training course to educate healthcare professionals on ways to effectively communicate and relate to HD patients and families. This course was thought to affect the quality of the care provided and, consequently, improve the quality of life of everyone involved.

**Materials and Methods:** The EHA implemented the training for Russian healthcare professionals over three Saturdays (12 hours) via Zoom. 20 HD experts from all over the world addressed topics such as verbal/non-verbal communication, HD-related communication changes or communication during genetic testing. Original learning formats were used, including the screening of interviews with HD families, group discussions or polls with instant feedback. To assess the “Let Us Talk” impact, the EHA created a structured online questionnaire about good and bad communication skills in clinical settings, which was administered at the beginning of the course and one week after its end. 20 behaviours were scored in terms of frequency, on a scale ranging from 1—Never to 5—Always. The results of the two measurements were analysed and compared.

**Results:** 111 Russian healthcare professionals attended the course, mainly neurologists (79.2%). 59 participants answered the questionnaire at baseline and 30 answered one month later.

Significant differences were found in some of the behaviours adopted before and after the training, namely a decreased use of medical jargon and a greater recognition of patients’ questions and concerns and of the importance of giving patients’ time to speak (Fig. 1).

**Fig. 1:**
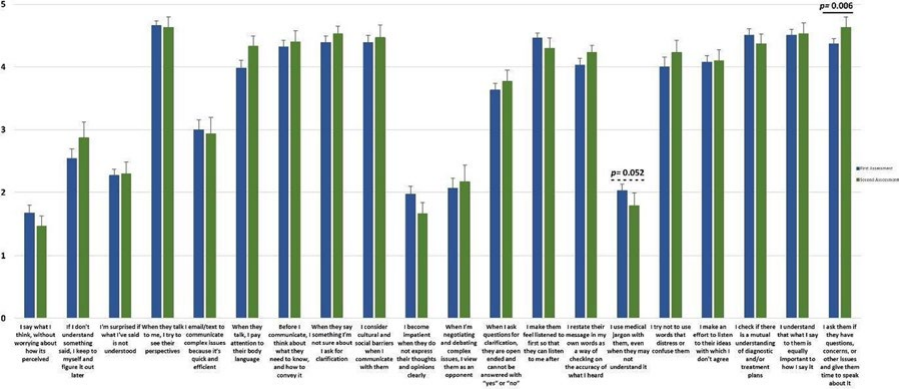
Communication Skills Questionnaire Results

**Conclusions:** “Let Us Talk” seems to have improved the communication skills of Russian healthcare professionals, who reported more appropriate behaviours towards HD families after the course. This training appears to be a good model for the EHA to replicate in other countries and enhance the doctor-HD patient relationship across Europe.

**Acknowledgements**

This work was done on behalf of the European Huntington Association (EHA)—Moving Forward Project. This work was partially funded by the European Federation of Neurological Associations (grant awardee for projects about Personalised Health and Social Care 2021), F. Hoffmann‐La Roche Ltd., Novartis and PTC Therapeutics.

## P3

### Innovating for people living with a rare disease: Why partnerships are key for the European OMP ecosystem

#### Christian Jervelund^1,*^, Julia S. Wahl^2^, Tuomas Haanperä^3^, Emil Löfroth^4^, Nikolaj Siersbæk^1^, Elisa Pau^2^, Laura Virtanen^1^, Toon Digneffe^5^ and Luana Banu^5^

##### ^1^Copenhagen Economics, Copenhagen, Denmark. ^2^Copenhagen Economics, Brussels, Belgium. ^3^Copenhagen Economics, Helsinki, Finland. ^4^Copenhagen Economics, Stockholm, Sweden. ^5^Takeda Pharmaceutical Company, Opfikon, Switzerland

*Corresponding author: cj@copenhageneconomics.com

*Orphanet Journal of Rare Diseases* 2023: P3

**Background:** The last two decades have seen many advancements in the development of orphan medicinal products (OMPs). Despite significant improvements, great unmet needs persist among rare disease patients. For example, approximately 95% of rare diseases, consisting mostly of the rarest of diseases, still lack authorised treatment altogether [1]. However, the unmet needs of rare disease patients and their caregivers extend beyond this, from the lack of transformative and curative treatment, over challenges concerning diagnosis and delayed and unequal access across Europe, to fragmented and inexperienced healthcare systems. Addressing unmet needs is challenging due to barriers along the entire OMP lifecycle, from basic research to patient access [2]. With the revision of the European OMP Regulation, Europe has a chance to review its policy framework and incentive environment with solutions to overcome these barriers.

**Materials and methods:** Industry expert interviews and desk research of existing initiatives were conducted to design implementable and barrier-specific solutions, based on an identification of their key enabling factors. A quantitative analysis was conducted using risk-adjusted net present value (rNPV) modelling of hypothetical orphan medicines to estimate the effect of possible revisions of the OMP Regulation and partnership-focused solutions on the incentives to invest. The analysis was based on publicly available data and data from a global pharmaceutical company.

**Results:** Adjusting current tools of the OMP Regulation, such as years of orphan market exclusivity, is in itself unlikely to have the desired effects required to address the unmet needs of patients with a rare disease today. The qualitative and quantitative analysis shows that partnership approaches rather than unilateral incentive-based solutions are needed to attract more development in rare diseases. For example, the quantitative analysis shows that while two additional years of market exclusivity (ME) for a hypothetical ultra-rare OMP will increase rNPV with 7%, a partnership solution will increase the rNPV by 21% (Fig. 1).

**Fig. 1:**
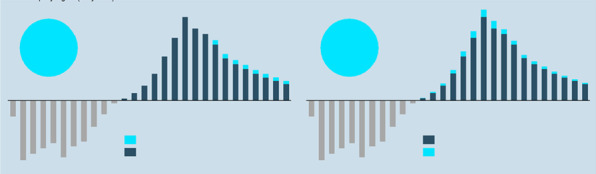
Effect of an increase in length of ME (left) and partnership solution (right) on the rNPV of a hypothetical ultra-rare OMP [3]. ME = market exclusivity, rNPV = risk-adjusted net present value

**Conclusions:** Seven recommendations for European policymakers are made, one of which includes evolving the current incentive framework of the OMP Regulation by recalibration of incentives, while the remaining six recommendations deploy partnership approaches to addressing unique barriers along the OMP lifecycle (Fig. 2).

**Fig. 2:**
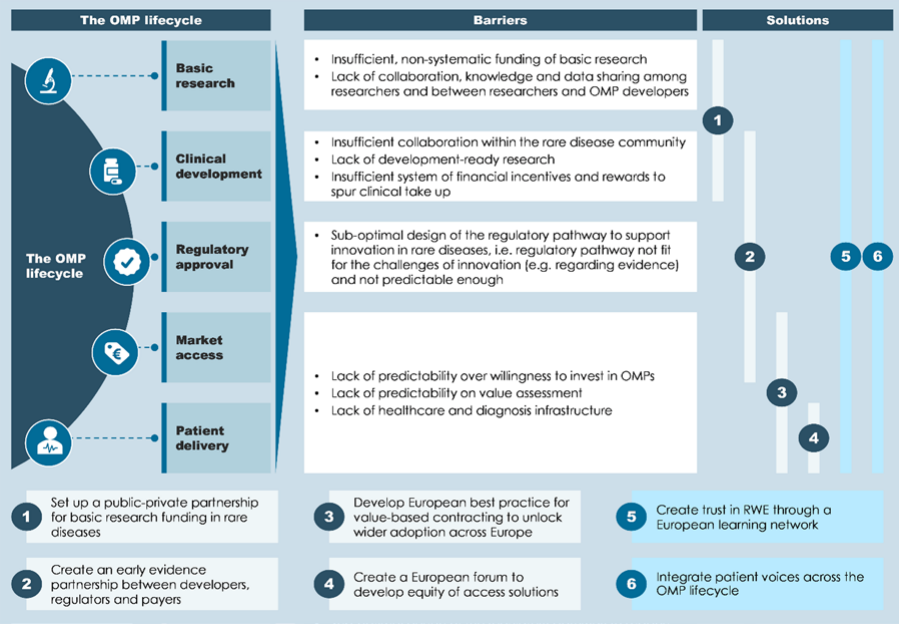
Six partnerships that can transform the OMP lifecycle. OMP = orphan medicinal product, RWE = real-world evidence. Builds on [2] and [3]

**Acknowledgements**

We thank industry experts for their participation in interviews.

**References**

1. European Commission. Joint evaluation of Regulation (EC) No 1901/2006 of the European Parliament and of the Council of 12 December 2006 on medicinal products for paediatric use and Regulation (EC) No 141/2000 of the European Parliament and of the Council of 16 December 1999 on orphan medicinal products [Internet]. 2020 Aug 11 [cited 2022 Jul 12]. Available from https://eur-lex.europa.eu/resource.html?uri=cellar:e9a9fff0-dbd9-11ea-adf7-01aa75ed71a1.0001.02/DOC_1&format=PDF.
2. Aartsma-Rus A, Dooms M, Le Cam Y, OD Expert Group, Copenhagen Economics. Orphan medicine incentives: how to address the unmet needs of rare disease patients by optimizing the European orphan medicinal product landscape guiding principles and policy proposals by the European expert group for orphan drug incentives (OD Expert Group). Front Pharmacol. 2021:12,744532.
3. Jervelund C, Wahl J S, Haanperä T, Löfroth E, Siersbæk N, Pau E, Virtanen L. Innovating for people living with a rare disease—Why partnerships are key for the European OMP ecosystem. 2021 Oct [cited 2022 Jul 13]. Available from https://copenhageneconomics.com/wp-content/uploads/2021/12/innovating-for-people-living-with-a-rare-disease_copenhagen-economics_2021.pdf.

## P4

### Collaborative project to improve access to genetic services in South Africa

#### Marianne CM Gomes^1,*^, Kelly du Plessis^1^, Helen L Malherbe^1,2^

##### ^1^Rare Diseases South Africa, Johannesburg, South Africa. ^2^Department of Biochemistry, Genetics and Microbiology, University of Pretoria, Pretoria, South Africa

*Corresponding author: genetics@rarediseases.co.za

*Orphanet Journal of Rare Diseases* 2023: P4

**Background:** Genetic services were first introduced into South Africa in the 1950’s. From 1960, as child mortality decreased and life expectancy at birth increased, congenital disorders (CD), and rare diseases (RD) emerged as a health care issue and countrywide consultation was undertaken to address them. However, the HIV/AIDS epidemic in the 1990’s effectively reversed these efforts, resulting in the neglect of genetic services. Today, comprehensive genetic services are available in only two of the nine provinces through three urban, academic centres—serviced by 13 practicing medical geneticists and 25 genetic counsellors for 62 million people. This is well below the recommended 31 medical geneticists and 107 genetic counsellors for the current population. This project aims to improve patient access to genetic services, particularly in provinces lacking these services.

**Methodology:** Rare Diseases South Africa (RDSA), an NPO that advocates for people living with CD/RD is partnering with the South African Society of Human Genetics (SASHG) to augment national genetic services and improve patient referral to relevant genetic specialists. This pilot, coordinated by RDSA, involves SASHG medical geneticists and genetic counsellors on a voluntary, rotational basis consulting with patients according to their geographical location, both in-person and virtually. Patients meeting defined criteria will be referred appropriately, either to mainstream genetic services already in existence or via the project-based services for urgent cases.

**Progress to date:** Criteria for patient referral, and a process flow have been developed and a call made to assist those patients on the RDSA membership network who had not previously received a genetic consultation. The pilot will be evaluated after six-months and revised as necessary. This approach offers an alternative, innovative approach to augment genetic services in South Africa and may be applicable to other developing countries.

**Acknowledgements**

Thank you to Dr K Fieggen, Dr C Spencer, Prof M Urban and Ms N Laing from SASHG for their input into the development of the project.

## P5

### Improved managed entry agreement negotiations and faster access to innovative therapies for rare disease patients: a value-based negotiation framework

#### Amanda Whittal^1,*^, Claudio Jommi^2^, Gérard De Pouvourville^3^, David Taylor^4^, Lieven Annemans^5^, Lies Schoonaert^6^, Sebastian Vermeersch^6^, Adam Hutchings^1^ and Julien Patris^7^

##### ^1^Dolon Ltd. 63 St Mary Axe EC3A 8AA, London, UK. ^2^Cergas (Centre for Research on Health and Social Care Management), SDA Bocconi School of Management, Bocconi University, Milan, Italy. ^3^Department of Economics, ESSEC Business School, Cergy-Pontoise, France. ^4^University College London, Gower Street, WC1E 6BT, London, UK. ^5^I-CHER, Ghent University, Ghent, Belgium. ^6^Hict, Gent, Belgium. Ottergemsesteenweg Zuid 808 B/354, 9000 Gent, Belgium. ^7^Alnylam Pharmaceuticals. Brussels, Belgium. Grafenauweg 4, 6300 Zug, Switzerland

*Corresponding author: amanda.whittal@dolon.com

*Orphanet Journal of Rare Diseases* 2023: P5

**Background:** Innovative therapies hold substantial potential for rare disease patients, yet these therapies often raise concerns around affordability and/or evidential uncertainties. In circumstances of high value and high uncertainty concerns, managed entry agreements (MEAs) can enable patient access to a treatment that would otherwise not be reimbursed because of such concerns [1]. However, barriers to MEA use have raised scepticism about their value, and differences in manufacturer and payer perspectives can lead to lengthy negotiations and delays in access [2–4].

A method is therefore needed to manage affordability and evidential uncertainty concerns, in order to accelerate access for rare disease patients to promising innovative therapies.

**Materials and methods:** An iterative process of scientific literature review and expert input was conducted to develop a conceptual framework. Subsequently, roundtable events were held in Belgium, The Netherlands, France and at the European level, for stakeholders to experience the framework and provide feedback on its practical usefulness.

**Results:** The value-based negotiation framework (VBNF) (Fig. 1) can facilitate faster, more structured MEA negotiations by (1) systematically identifying and prioritising manufacturer and payer concerns about a product, and (2) supporting the selection of a mutually acceptable combination of MEA terms that can best address priority concerns, with the lowest possible implementation burden.

**Fig. 1:**
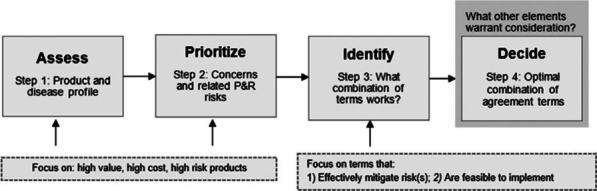
Stepwise value-based negotiation framework for innovative therapies. Previously published [6]

Roundtable feedback was constructive and positive, enabling further refinement of the framework and generating interest in testing it in practice.

**Conclusions:** The VBNF is a step toward supporting payers and manufacturers to engage in more structured and efficient MEA negotiations for innovative therapies. It can help balance the needs of both negotiating parties, while coming to a mutually acceptable agreement—and therefore patient access to innovative products—more quickly. Despite the conceptual basis, the framework has corresponding practical tools to enable its application in practice. It is designed to be adaptable to varying jurisdiction-specific systems or even cross-country collaborations. The next steps are to continue to introduce the framework concept, as well as apply it in practical settings.

**References**

1. Annemans L, Pani L. Dynamic outcomes based approaches to pricing and reimbursement of innovative medicines. National Institute for Health and Disability Insurance (RIZIV-INAMI) [Accessed 6 August 2021]. Available at: https://www.eurordis.org/sites/default/files/FIPRA.pdf.2017
2. Dabbous M, Chachoua L, Caban A, Toumi M. Managed entry agreements: policy analysis from the European perspective. Value in Health. 2020 Apr 1;23(4):425–33.
3. Klemp M, Frønsdal KB, Facey K. What principles should govern the use of managed entry agreements?. International journal of technology assessment in health care. 2011 Jan;27(1):77–83.
4. A Vreman R, F Broekhoff T, GM Leufkens H, K Mantel-Teeuwisse A, G Goettsch W. Application of managed entry agreements for innovative therapies in different settings and combinations: A feasibility analysis. International Journal of Environmental Research and Public Health. 2020 Nov;17(22):8309.
5. Wenzl M, Chapman S. Performance-based managed entry agreements for new medicines in OECD countries and EU member states: How they work and possible improvements going forward.
6. Whittal A, Jommi C, De Pouvourville G, Taylor D, Annemans L. (2022) Facilitating More Efficient Negotiations for Innovative Therapies: A Value-Based Negotiation FrameworkInternational Journal of Technology Assessment in Health Care, Vol 38(1) https://doi.org/10.1017/S0266462322000095

## P6

### Facilitating the creation of a bottom-up European Network of Sickle Cell Disease Patients Organizations

#### Mariangela Pellegrini^1,*^, Angelo Loris Brunetta^2^, Ariane Weinman^3^, Beatrice Gulbis^4^, Raffaella Colombatti^5^, Mariane de Montalembert^6^, Baba Psalm Duniya Inusa^7^, Noémi BA Roy^8^, Pierre Fenaux^1^, Maria del Mar Manu Pereira^9^

##### ^1^Assistance Publique—Hôpitaux de Paris, Hôpital Saint Louis, Paris, France. Member of European Reference Network on Rare Hematological Diseases. ^2^ePAG ERN-EuroBloodNet for Red Blood Cell Defects Subnetwork, Thalassaemia International Federation. ^3^EURORDIS, Rare Diseases Europe, Paris, France. ^4^Hôpital Erasme/LHUB-ULB, Brussels, Belgium. Member of European Reference Network on Rare Hematological Diseases. ^5^Pediatric Hematology Oncology, Department of Woman’s and Child’s Health, University of Padova, Italy. Member of European Reference Network on Rare Hematological Diseases. ^6^Assistance Publique—Hôpitaux de Paris, Hôpital Necker, Paris, France. Member of European Reference Network on Rare Hematological Diseases. ^7^Paediatric Haematology, Evelina London Children’s Hospital, Guy's and St Thomas' NHS Foundation Trust, London, UK. ^8^Oxford University Hospitals NHS Trust, London, UK. ^9^Vall d'Hebron Research Institute/Vall d’Hebron University Hospital, Barcelona, Spain Member of European Reference Network on Rare Hematological Diseases

*Corresponding author: mariangela.pellegrini@aphp.fr

*Orphanet Journal of Rare Diseases* 2023: P6

**Background:** Sickle Cell Disease (SCD) is an inherited disorder of the red blood cells. Being a lifelong chronic condition, SCD can lead to disability and/or premature death in its acute forms. It is traditionally endemic in African and Middle East countries [1, 2, 3,] but their frequency has increased in Europe due to migration and mobility flows [4, 5, 1, 6, 7, 8]. Those patients often face integration difficulties with less facility to access care services [9].

**Material and methods:** Since 2017 the ERN-EuroBloodNet with EURORDIS is facilitating the creation of a bottom-up European Network of SCD Patients Organizations, because the existing patients’ organizations were scattered across Europe and not often worked synergically to improve patients’ quality of life. The main objective of this project is to provide a centralized European point of contact for patients and caregivers, encouraging the establishment of national patients’ association in those countries where patients’ associations do not exist yet. As language barriers proved to be an issue for patients with different cultural backgrounds, national meetings with local SCD Patient Organizations were organized in Belgium, France, Italy, Spain & Portugal to bring together the existing patient organizations or patients’ groups to raise awareness about the activities of the ERN-EuroBloodNet and EURORDIS. Following these meetings, in 2019, within the context of ASCAT (Annual Congress on Sickle Cell and Thalassemia), a first educational session has been organized from the ERN-EuroBloodNet to discuss with SCD patient representatives the "Top 10" priorities on research from their perspective. Following events took place remotely in 2020 and in January 2022, next one will be held in October 2022. A kick-off meeting with all the SCD Patients’ representatives, supported by the ERN, was held in December 2021, for evaluating the maturity of the project implemented and identifying initial potential actions to be carried out by the group of Patients’ Organisations and to identify potential SCD patient candidates for becoming ePAGs.

**Results:** More than 50 patient organizations have been involved in actions implemented by the ERN-EuroBloodNet. At least two National patients’ representatives have been identified for Italy, France, Spain, Portugal and Belgium (Fig. 1).

**Fig. 1:**
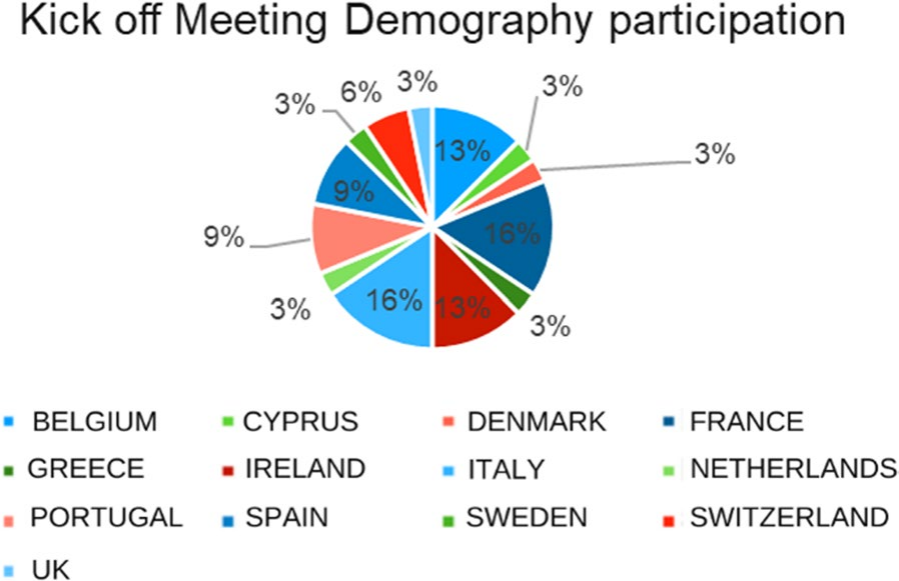
Participants demography of the Kick off meeting

**Conclusions:** As the SCD patients’ organizations are not familiar to work in synergy at European level, the creation of a Network allows the introduction of representatives in the advocacy field, therefore initiating them to be part of the ePAGs and ERNs community.

**Acknowledgements**

This work is generated within the European Reference Network on Rare Hematological Diseases (ERN-EuroBloodNet). FPA 739541.

**References**

1. Piel, F.B., Steinberg, M.H. & Rees, D.C. (2017) Sickle cell disease. New England Journal of Medicine, 376, 1561–1573. Crossref CAS PubMed Web of Science®Google Scholar
2. Serjeant, G. (2017) World sickle cell day: lessons for India. Indian Journal of Medical Research, 145, 705–707. Crossref PubMed Web of Science®Google Scholar
3. Ware, R.E., de Montalembert, M., Tshilolo, L. & Abboud, M.R. (2017) Sickle cell disease. Lancet, 390, 311–323. Crossref PubMed Web of Science®Google Scholar
4. Roberts, I. & de Montalembert, M. (2007) Sickle cell disease as a paradigm of immigration hematology: new challenges for hematologists in Europe. Haematologica, 92, 865–871. Crossref PubMed Web of Science®Google Scholar
5. Mañú Pereira M, Corrons JL. Neonatal haemoglobinopathy screening in Spain. J Clin Pathol. 2009 Jan;62(1):22–5. https://doi.org/10.1136/jcp.2008.058834. PMID: 19103853.
6. Cortes‐Castell, E., Palazon‐Bru, A., Pla, C., Goicoechea, M., Rizo‐Baeza, M.M., Juste, M. & Gil‐Guillen, V.F. (2017) Impact of prematurity and immigration on neonatal screening for sickle cell disease. PLoS ONE, 12, e0171604. Crossref PubMed Web of Science®Google Scholar
7. Inusa, B.P.D. & Colombatti, R. (2017) European migration crises: the role of national hemoglobinopathy registries in improving patient access to care. Pediatric Blood & Cancer, 64, e26515. Wiley Online Library Web of Science®Google Scholar
8. Kunz, J.B., Cario, H., Grosse, R., Jarisch, A., Lobitz, S. & Kulozik, A.E. (2017) The epidemiology of sickle cell disease in Germany following recent large‐scale immigration. Pediatric Blood & Cancer, 64, e26550. Wiley Online Library CAS Web of Science®Google Scholar
9. de Montalembert, M., Ferster, A., Colombatti, R., Rees, D.C. & Gulbis, B. (2011) ENERCA clinical recommendations for disease management and prevention of complications of sickle cell disease in children. American Journal of Hematology, 86, 72–75. Wiley Online Library PubMed Web of Science®Google Scholar

## P7

### Leading the change by prioritising patients' needs: Effèmerides

#### Simona Bellagambi^1,*^, Annalisa Scopinaro^1,*^

##### ^1^UNIAMO- Rare Diseases Italy, Rome, Italy

*Corresponding author: bellagambi.estero@uniamo.org, presidente@uniamo.org

*Orphanet Journal of Rare Diseases* 2023: P7

**Purpose:** Develop shared positions on specific issues between patient representatives and institutions, to push policymakers to take decisions based on the outcomes and highlights of the meetings.

**Target group:** Parliamentarians, technical bodies of Ministries, National and Regional Institutions, other stakeholders.

**Methodology:** Thematic online meetings, two for each topic, were held on the European Regulation on orphan drugs, ATMP, job inclusion for PLWRD, Serious Disabilities with the representatives of patient organizations, sector experts, public and private stakeholders, a group coordinator. The work involved identifying the existing literature and legislation, current criticalities and possible solutions, and the right interlocutors in charge of overcoming the critical issues.

Ahead of the first meeting the relevant material was disseminated; the final report shared and amended by the participants before its publication in the editorial series.

**Results:** The result of each table was condensed into an editorial series created ad hoc, the Effèmerides of UNIAMO, launched during the celebrations for the Rare Disease Day.

The Serious Disability position paper was used by the Minister of Labor and Social Policy for some general measures; the position on the job inclusion served to respond to the public consultation of the Ministry and led to a hearing with the technical bodies of the Ministry; the position on the European Regulation was widely shared by the participants during various webinars / conferences and meetings.

The other outcomes were presented to the competent Ministers for Health and Disability and also represented the basis for responding to public calls on individual topics.

Overall, about 40 PO representatives and 30 different stakeholder representatives participated in these meetings. In the dedicated public event the audience was about 70 people.

**Conclusions:** Small working groups demonstrated to be an efficient way to concretely address relevant topics thus we’ll continue to hold these meetings on different relevant issues either face to face or hybrid meetings. The editorial series will become a reliable source of information and a basis for policy makers.

## P8

### Third French National Plan for Rare Diseases: establishment of a diagnosis observatory

#### Sarah Otmani^1,*^, Arnaud Sandrin^1^, BNDMR operational Unit^1^, Anne-Sophie Lapointe^2^, Céline Angin^1^

##### ^1^French national rare disease registry (BNDMR), AP-HP, Paris, France. ^2^French Ministry for Solidarity and Health, General Directorate of Health Care Provider (DGOS), Paris, France

*Corresponding author: contact.bndmr@aphp.fr

*Orphanet Journal of Rare Diseases* 2023: P8

Reducing diagnostic delays and undiagnosed diseases is one of the French National Plan for Rare Diseases 3 (PNMR3) [https://solidarites-sante.gouv.fr/IMG/pdf/pnmr3_-_en.pdf] priorities (action1.7) funded by the General Directorate of Health Care Provider (DGOS), linked to the Ministry of Health and Prevention, to the tune of €3 million per year for the duration of the plan (2018–2022).

To achieve this objective, one strategy is to reassess the cases of currently undiagnosed patients in order to obtain a diagnosis in light of scientific advances. This will reduce the risks of loss of opportunity in terms of diagnosis and potentially treatment. To help the Rare Disease Reference Centres (CRMR) in this task and to monitor the progress of diagnoses at the national level, a dynamic database is being set up from the French national rare diseases registry (BNDMR) [https://www.bndmr.fr/] with the support of the 23 national rare disease clinical networks (FSMRs) [https://www.filieresmaladiesrares.fr/].

The BNDMR, created as part of the PNMR 2, collects from all expert centers on rare diseases a minimal data set (MDS), either from a web app (BaMaRa) or from the electronic health record (EHR). The FSMRs are committed to entering this MDS in all the CRMR, and to support them towards better data quality. Some also want to collect additional data in BaMaRa for patients without a diagnosis. More than 50 people (especially clinical research associates and technicians) were recruited in addition to the staff mobilised on these actions.

A diagnosis observatory [https://solidarites-sante.gouv.fr/soins-et-maladies/prises-en-charge-specialisees/maladies-rares/article/l-observatoire-du-diagnostic] has been set up to follow the diagnostic delay and undiagnosed diseases situations and to assess the impact of the measures taken. It is based in particular on annual indicators from the database and aims to ensure consistency of practices and the incorporation of recent discoveries in medical care, in particular those resulting from the France Genomic Medicine 2025 plan [https://pfmg2025.aviesan.fr/en/].

## P9

### IGA Volunteer programme: Building an international patient group: achieving a global reach with limited resources

#### Vesna Aleksovska^1,*^, Tanya Collin-Histed^2,*^

##### ^1^International Gaucher Alliance, Dursley, Gloucestershire, United Kingdom. ^2^International Gauc sec4her Alliance, Dursley, Gloucestershire, United Kingdom

*Corresponding author: vesna@gaucheralliance.org, tanya@gaucheralliance.org

*Orphanet Journal of Rare Diseases* 2023: P9

The overall goal of the volunteer program is to support the work of IGA and enable us to meet the diverse needs of our global community. Through increasing the numbers of volunteers, IGA is gathering more experience, perspectives from different countries and cultures, new ideas, and new ways on how to improve quality of life of families with Gaucher disease. The programme was developed to provide a structure and invests in the volunteers through providing training and supervision.

The Volunteer programme has its own strategic plan, in accordance with the IGA strategic plan. Also, we developed additional documents that support the program such as volunteer job description, time sheet, recognition, and support of volunteers. All volunteers were interviewed and went through orientation before they start volunteering and each one had a mentor or a project lead to support them. Volunteers have regular meetings with the volunteer coordinator, and they are involved in improvement of the volunteer programme through feedback and evaluation questionnaire. Additional training and workshops are available for volunteers to attend and expand their skills and knowledge.

**Results:** In 2021 and 2022 IGA has had support from 41 volunteers, and the number is increasing.

- Volunteers were involved in **11 projects**.
- Volunteers come from **34 different countries**
- Most of the volunteers (around 70%) were recruited through advertisement on social media and some of them were directly invited to join in projects and activities in IGA.
- In 2021 we had around **1400 volunteer hours**.
- The Regional Manager Programme (increasing our footprint into new countries) and the GARDIAN Champions Raising awareness of our nGD registry) program is completely driven by volunteers—without them both programmes would not have been possible. Now a total of 18 people is involved in them, and this number is growing.

**Conclusion:** The Volunteer Programme is a support in many ways for IGA.

- It is an established system to recruit and retain volunteers
- It provides future recruitment of board members from existing volunteer pool of accomplished volunteers who want to be more involved in IGA work
- It enables the IGA to have an increased impact through the realization of more projects
- There is less burden of tasks and projects for board members which allows them to focus on strategy and leading the organization forward
- IGA gets an increased skill set
- Expanded community support is noticeable in IGA projects.

## P10

### Work participation in adults with rare diseases—a systematic scoping review of relevant research

#### Gry Velvin^1,*^, Brede Dammann^1^, Trond Haagensen^1^, Heidi Johansen^1^ and Trine Bathen^1^

##### ^1^TRS Resource Centre for Rare Disorders, Sunnaas Rehabilitation Hospital, Nesoddtangen Norway

*Corresponding author: Gry.Velvin@sunnaas.no

*Orphanet Journal of Rare Diseases* 2023: P10

**Background:** Work participation is a social determinant of health and important for understanding health behaviours, health outcomes and quality of life among people with rare diseases. It is an under-recognized and under-researched aspect in many rare diseases. The purpose of this study was to map existing research on work participation in rare genetic diseases and some other selected rare diseases, identify research gaps, and point to research agendas.

**Methods:** A scoping review methodology was applied. We conducted systematic searches in September 2021 in ten bibliographic databases. References were sorted and assessed for inclusion using EndNote and Rayyan software. Data were extracted on the main research questions on work participation in adults with rare diseases.

**Results:** A total of 166 papers on 36 different diseases fulfilled our inclusion criteria: 7 reviews and 159 primary articles. Only 34 articles had major focus on work participation. There were large differences in the number of studies on the different rare diseases. Only two diseases had more than 20 articles addressing work participation, most diseases had only one or two articles. Nearly all articles (96%) reported information about prevalence of work participation, and more than half also included information about factors associated with work participation and work disability. Some studies also indicated particular challenges related to having a rare disease. Due to differences in methodology, culture and respondents, comparison between and within diseases are difficult.

**Conclusion:** While studies indicate high prevalence of work-disability in many patients with rare diseases, the research is scarce and fragmented. There is a need for more research on work participation. This includes both primary and secondary research, including all types of research questions: prevalence, associations, patients’ experiences and views, validation of outcome measures and effect of different intervention programs. Information on the unique challenges of living with the different rare diseases is important for health and welfare systems to better facilitate vocational situation. In addition, the changing nature of work in the digital age may also open up new possibilities and may reduce the inequality experienced by people with rare diseases.

**Acknowledgements**

The authors want to thank the TRS National Resource Centre for Rare Disorders, and special thanks to the members of our research group Amy Østertun Geirdal and Hilde Strømme for important academic contributions.

## P11

### Using ADDIE learning model to design a multidisciplinary educational program centred on rare diseases addressed to high-school students in Romania

#### Ioana C Stănculescu^1,*^, Zina Barabas-Cuzmici^2^, Andreea Cătană^2^, Eleonora Dronca^2^, Ana C Zdrenghea^3^, Monica A Mager^4,5^, Steluța Palade^5^

##### ^1^Babeș-Bolyai University, Cluj-Napoca, Romania. ^2^Department of Molecular Sciences, Iuliu Hațieganu University of Medicine and Pharmacy, Cluj-Napoca, Romania. ^3^Cluj County Hospital, Cluj-Napoca, Romania. ^4^Department of Neurosciences, Iuliu Hațieganu University of Medicine and Pharmacy, Cluj-Napoca, Romania. ^5^Children’s Emergency Hospital, Clinic of Paediatric Neurology, Cluj-Napoca, Romania

*Corresponding author: oanablaga@gmail.com

*Orphanet Journal of Rare Diseases* 2023: P11

Knowledge on rare diseases in general population has been shown to be limited [1] across the world. This has a profound influence on people’s attitudes towards the affected patients. Romania has a low level of health literacy [2], especially in the rural areas where a concerningly high percentage of population lacks basic information on health topics. The combination of this low level of health literacy and the popular beliefs that prevail especially in the rural areas, results in stigma and lack of inclusion for the people affected by rare diseases. Education is a good tool to provide the young generation with quality information that will efficiently shift their perspective in the field of health education.

The authors plan to develop a course on rare diseases adapted for high-school students. The protocol of the course will be designed using the ADDIE learning system, a versatile model that has been proven to be successful in medical education before [3–7]. The course will begin with a complex analysis of the students, their needs, their preferred learning method, and the learning environment. Based on this step, the design and development phases will help us draw the blueprint of the modules and the lessons and create the learning materials. The course is designed as an 8-week multidisciplinary program, involving the participation of teachers, doctors, and representatives of patients’ organizations. Once the design is configured and the materials prepared, the course can be implemented according to the schedule. At the end of the program, summative and formative evaluations will be performed.

The expected results are an increase in the knowledge of the young population on the topic of rare diseases and a shift in their approach of affected patients leading to a reduction in social inequalities.

**References**

1. Ramalle-Gómara E, Ruiz E, Quiñones C, Andrés S, Iruzubieta J, Gil-de-Gómez J. General knowledge and opinion of future health care and non-health care professionals on rare diseases. J Eval Clin Pract. 2015;21(2):198–201.
2. Pop OM, Brînzaniuc A, Şirlincan EO, Baba CO, Cherecheş RM. Assessing health literacy in rural settings: a pilot study in rural areas of Cluj County, Romania. Global Health Promotion. 2013;20(4):35–43.
3. Patel SR, Margolies PJ, Covell NH, Lipscomb C, Dixon LB. Using Instructional Design, Analyze, Design, Develop, Implement, and Evaluate, to Develop e-Learning Modules to Disseminate Supported Employment for Community Behavioral Health Treatment Programs in New York State. Front Public Health. 2018;6:113.
4. Battles JB. Improving patient safety by instructional systems design. Qual Saf Health Care. 2006;15 Suppl 1(Suppl 1):i25–i29.
5. Verger S, Negre F, Fernández-Hawrylak M, Paz-Lourido B. The Impact of the Coordination between Healthcare and Educational Personnel on the Health and Inclusion of Children and Adolescents with Rare Diseases. Int J Environ Res Public Health. 2021;18(12):6538.
6. Walkowiak D, Domaradzki J. Are rare diseases overlooked by medical education? Awareness of rare diseases among physicians in Poland: an explanatory study. Orphanet J Rare Dis. 2021;16(1):400.
7. Fernandes RAML, de Oliveira Lima JT, da Silva BH, Sales MJT, de Orange FA. Development, implementation, and evaluation of a management specialization course in oncology using blended learning. BMC Med Educ. 2020;20(1):37.

## P12

### The Spanish Genomic Medicine program (IMPaCT-GENóMICA): reducing diagnostic inequalities for rare diseases patients

#### Pablo Lapunzina^1,2^, Carmen Ayuso^2,3^, Gabriel Capellá^4,5^, Ivo Gut^6^, Adrián Llerena^7^, Juan Luque^2^, Esther Sande^2,9^, Beatriz Sobrino^2,9,10^, Yolanda Benítez^2^, Lucía Pérez de Ayala^2^, Beatriz Morte^2^, Beatriz Gómez^2^, Ángel Carracedo^2,8,9,10,*^

##### ^1^Instituto de Genética Médica y Molecular (INGEMM)-IdiPAZ, Hospital Universitario La Paz-UAM, Madrid, Spain. ^2^Centro de Investigación Biomédica en Red de Enfermedades Raras (CIBERER-ISCIII), Madrid, Spain. ^3^Department of Genetics, Instituto de Investigación Sanitaria-Fundación Jiménez Díaz University Hospital (IIS-FJD, UAM), Madrid, Spain. ^4^Catalan Institute of Oncology, IDIBELL, Hospitalet de Llobregat, Barcelona, Spain. ^5^Centro de Investigación Biomédica en Red de Cáncer (CIBERONC-ISCIII), Barcelona, Spain. ^6^Centro Nacional de Análisis Genómico (CNAG), Barcelona, Spain. ^7^Clinical Research Center (CICAB), Extremadura University Hospital and Medical School, Badajoz, Spain. ^8^Universidade de Santiago de Compostela (USC), Santiago de Compostela, Spain. ^9^Instituto de Investigación Sanitaria de Santiago (IDIS), Santiago de Compostela, Spain. ^10^Fundación Pública Galega de Medicina Xenómica (FPGMX), SERGAS, Santiago de Compostela, Spain

*Corresponding author: genomica.impact@ciberisciii.es

*Orphanet Journal of Rare Diseases* 2023: P12

The Genomic Medicine program (IMPaCT-GENóMICA) is one of the three programs that configure the Precision Medicine Infrastructure associated with Science and Technology (IMPaCT)], start up through the Strategic Action in Health 2017-2020 of Health Institute Carlos III (ISCIII) [2], belonging to the Spanish Ministry of Science and Innovation.

IMPaCT GENóMICA, with a budget of 7,24 M€, is coordinated by Dr. Carracedo from the Center for Biomedical Network Research on Rare Diseases (CIBERER) [3]. It promotes the implementation of a collaborative structure (Fig. 1) distributed across nodes (currently three) for high complexity genetic studies and a network of clinical, geneticists and molecular experts. It is madding up of 45 groups from 25 hospitals and 13 research centers or universities, and more than 300 researchers distributed all over the country. It is coordinated with the national health system and the Ministry of Health.

**Fig. 1:**
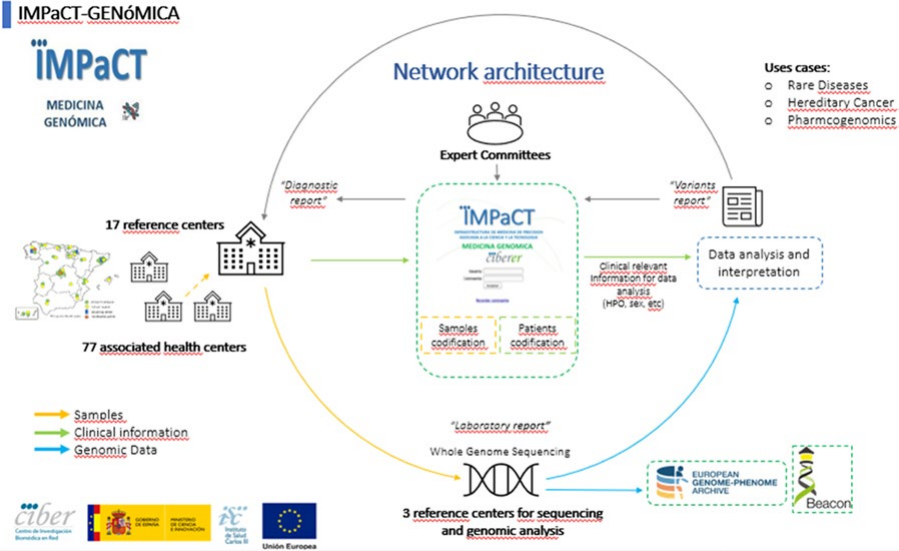
Network architecture

The main objective of this program is to provide the national health system this collaborative structure for the implementation of Genomic Medicine, so that patients that do not have a certain genetic diagnosis, after applying the established assistance diagnosed protocols, could access to experimental 'omics' diagnostic technologies with fairness and adequate response times.

The program also obtains genomic data that can be used in research, improves the analysis capabilities of the infrastructure, and contributes to the European initiative “1+ Million Genomes (1+MG)” [4].

IMPaCT-GENóMICA works on three use cases to achieve its objectives: rare diseases, hereditary cancer and pharmacogenomics and population genomics.

Especially for rare diseases, due to the great complexity of these pathologies, the step forward in the diagnosis needs an up-to-date knowledge and the application of the latest techniques, which are not always available. The networks and flows provided by IMPaCT-GENóMICA will improve the diagnosis of rare diseases in the national health system in an equitable manner throughout the territory.

**References**

1. Precision Medicine Infrastructure associated with Science and Technology [Internet]. Madrid, Health Institute Carlos III; [cited 2022 Jul 18]. Available from: https://www.isciii.es/QueHacemos/Financiacion/IMPaCT/Paginas/Plan.aspx
2. Health Institute Carlos III (ISCIII) [Internet]. Madrid, Instituto de Salud Carlos III; [cited 2022 Jul 18]. Available from: https://eng.isciii.es/eng.isciii.es/Paginas/Inicio.html
3. Center for Biomedical Network Research on Rare Diseases (CIBERER) [Internet]. Madrid, Instituto de Salud Carlos III; [cited 2022 Jul 18]. Available from: https://www.ciberer.es/en
4. 1 + Million Genomes (1 + MG) [Internet]. [cited 2022 Jul 18]. Available from: https://b1mg-project.eu/

## P13

### Exploring the feasibility of setting-up Advisory Committees for Therapeutics (ACTs) in rare diseases

#### Joanne Lee^1,*^, Cathy Turner ^1^, Victoria Hedley^1^, Volker Straub^1^

##### ^1^John Walton Muscular Dystrophy Research Centre, Newcastle University, Newcastle upon Tyne, UK

*Corresponding author: joanne.lee@ncl.ac.uk

*Orphanet Journal of Rare Diseases* 2023: P13

Within the European Joint Programme for Rare Diseases (EJP RD) [https://www.ejprarediseases.org], we have developed a toolkit to support disease communities to replicate the TREAT-NMD Advisory Committee for Therapeutics (TACT) model [https://treat-nmd.org/tact-treat-nmd-advisory-committee-for-therapeutics/tact-overview/]. TACT has been successful in the neuromuscular field for over 10 years and has reviewed over 70 applications for advice on the translational and development pathway of therapeutic programs in neuromuscular diseases. An Advisory Committee for Therapeutics (ACT) toolkit was created to provide procedural advice on how to set-up a committee of academic and industry drug development experts, including patients and patient representatives, to provide independent and objective advice on specific drug development programmes. The toolkit will soon be a freely available resource in the EJP RD Innovation Management Toolbox [https://imt.ejprarediseases.org].

An important aim of EJP RD is to identify good practices and support adoption in other communities. In the spirit of this, and to complement the toolkit we created, we applied to the EJP RD ERN training call for funding to hold a workshop designed to communicate the benefits of the model and to discuss the feasibility of ERN (European Reference Network) communities adopting the ACT model. This funding call was selected because strategic oversight is needed to expand the ACT model into other rare disease communities. The thematic groupings of the ERNs provide a logical framework to set-up an ACT in other rare diseases.

Representatives of 11 ERNs (a mixture of clinicians, researchers, and ePAGs) registered for this workshop. It introduced the concept of the ACT model, presented lessons learned from the neuromuscular field and facilitated break-out groups to discuss the feasibility of adopting the model within participants’ ERNs. A post-workshop survey was sent out and 11 out of the 16 participants said that they expect to explore the possibility of establishing an ACT within their ERN. The workshop organisers are now following up with participants and providing advice and support to implement the model in new fields using the ACT toolkit. This will have a major added value to rare disease research, by optimising the chances of success of planned clinical trials.

**Acknowledgements**

This initiative has received funding from the European Union's Horizon 2020 research and innovation programme under grant agreement N°825575.
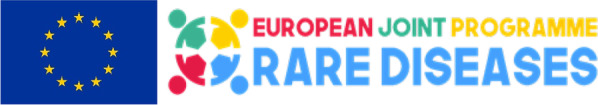


The Advisory Committee for Therapeutics (ACT) model is based on the TREAT-NMD Advisory Committee for Therapeutics (TACT), which has been successful in the neuromuscular field. TACT was developed through the neuromuscular network TREAT-NMD [https://treat-nmd.org], using funding granted through the European Union Framework Programme 6 to build a network of excellence.

## P14

### COMMUNITY NURSES AND CASE MANAGEMENT FOR RARE DISEASES IN ROMANIA

#### Lidia Onofrei^1^, Dorica Dan^2,3,4^, Birutė Tumienė^6^, Nicoleta Andreescu^1,3,5^, Alexandra Dan^2,3,4^, Maria Acaraliței^3,4^, Ramona Mocuțiu^3,4^, Maria Puiu^1,2,3,5^

##### ^1^Center of Genomics, Victor Babes University of Medicine and Pharmacy, Timisoara, Genetics Department, Timișoara, Romania. ^2^Romanian National Alliance for Rare Diseases, Zalau, Romania. ^3^Ro-NMCA-ID- ITHACA ERN, Louis Turcanu Pediatric Emergency Hospital Timisoara, Timisoara, Romania. ^4^Romanian Prader Willi Association (NoRo Center for RD), Zalău, Sălaj, România. ^5^Regional Centre for Medical Genetics Timis1 – Center of Genomics, Victor Babes University of Medicine and Pharmacy, Timisoara, Genetics Department, Timișoara, Romania. ^6^Vilnius University, Faculty of Medicine, Institute of Biomedical Sciences, Vilnius, Lithuania

*Orphanet Journal of Rare Diseases 2023, supp P14*

**Summary and methodology:** Romanian Prader Willi Association- RPWA, through NoRo Center have implemented in partnership with Eurordis and other partners the INNOVCare project during 2015 -2018, developed and tested a holistic care pathway to strengthen the medical, social, and educational services. To upscale our experience at national level, we identified that community nurses have the background and could support even the most isolated patients living with rare diseases in Romania if they get trained.

A partnership with Ministry of Health has been signed in April 2021 to train community nurses on case management for rare diseases. The methodology used in our training was ECHO (Extension for Community Healthcare Outcomes). It is an evidence-based professional development approach that can help nurses to stay current and apply new knowledge to practice ECHO: A Model for Professional Development in Nursing Through Learning Networks—https://doi.org/10.3928/00220124-20210315-09

We connected the community nurses with experts from the 31 centers of expertise accredited for rare diseases in Romania and facilitated their communication in order to structure together the patients’ journey and reduce the waiting time for patients until they get the proper diagnosis and care.

To address the need for integrated care we organized our advocacy activity around the nine pillars of integrated care identified by IFIC: Nine Pillars of Integrated Care—IFIC (integratedcarefoundation.org)—https://integratedcarefoundation.org/nine-pillars-of-integrated-care#1589383665414-08104e53-13cd

**Results:** Based on the partnership between the Ministry of Health, Romanian Prader Willi Association, and Romanian National Alliance for Rare Diseases, 390 out of 1850 community nurses hired at national level have been trained up to date.

According to the national platform for community nursing amcmsr.gov.ro, 937 of patients with rare diseases, 498 males and 439 females benefited from 3824 visits and interventions. A survey was applied at the end of each training, and we got the following results 17,51% of the trained community nurses declared that they have included new patients with RD during the training, 95,6% declared that the information provided was very useful and 92.6% considered that coordinating patients with Centers of Expertise is extremely useful.

**Conclusions:** To improve access to diagnostic and care for patients with rare diseases in Romania we will continue our advocacy for integrated care, our trainings for community nurses and develop a digital tool for the case management, to implement a more efficient monitoring and virtual care coordination of the patients to reduce the waiting time for diagnosis and care.

## P15

### Duchenne Map: Connecting all stakeholders in the global dystrophinopathy community, to advance research, therapy development, and healthcare decision-making

#### Katerina Tzima^1,*^, Suzie-Ann Bakker^1,*^, Paraskevi Sakellariou^1^, George Paliouras^1^

##### ^1^Duchenne Data Foundation, Veenendaal, The Netherlands

*Corresponding author: katerina@duchennedatafoundation.org, suzieann@duchennedatafoundation.org

*Orphanet Journal of Rare Diseases* 2023: P15

**The problem:** Fragmentation, duplication of effort and lack of reliable information are problems that all rare disease communities face. These factors may significantly hinder access to treatment, recruitment to clinical trials, eventually negatively affecting the development of new therapies, having a direct effect on the quality of life and care of people living with dystrophinopathies and their families [1].

**The solution:** The Duchenne Data Foundation has developed an online platform, the Duchenne Map (Fig. 1) [2], to make relevant and valid information accessible and connect patients with their local patient organizations, healthcare providers, care centers, researchers, research institutions and companies.

**Fig. 1:**
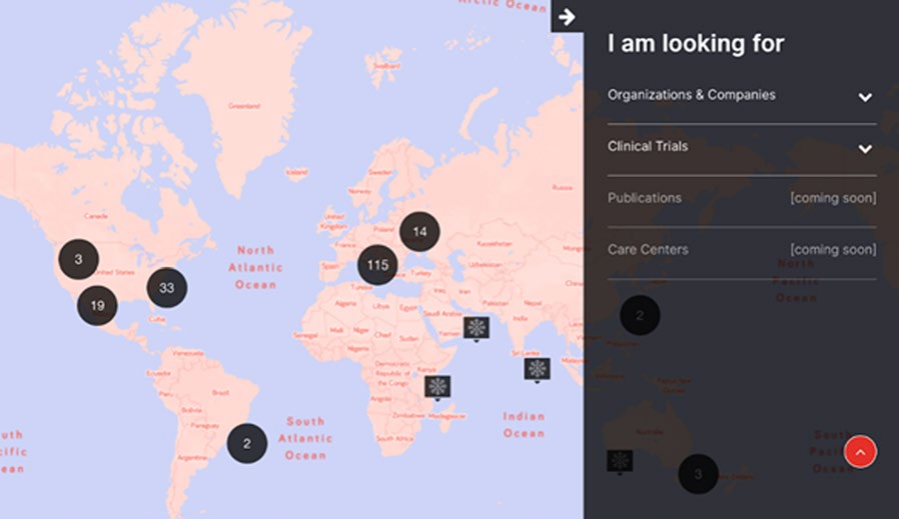
Overview of Duchenne Map for users

**How it works:** Users register on the Duchenne Map and complete their profile. A double verification process (machine process) and the validation process (human process) is in place to confirm the authenticity of the user. Once registered and validated, users can browse the Duchenne Map and find relevant and reliable information on multiple stakeholders in the Duchenne community at a global level, as well as research projects and clinical trials in their geographical area.

**Services:** Currently, registered users can find:

- 50 Patient Organizations, 5 Companies, 137 Clinical Trials
- A personalized clinical trial filter function (Fig. 2). Registered users can search recruiting clinical trials in their area according to age, use of corticosteroids and ambulation status.
- Additional filters allowing to search for recruiting clinical trials based on the mutation type and find care centers offering specific services are currently being implemented.

**Fig. 2:**
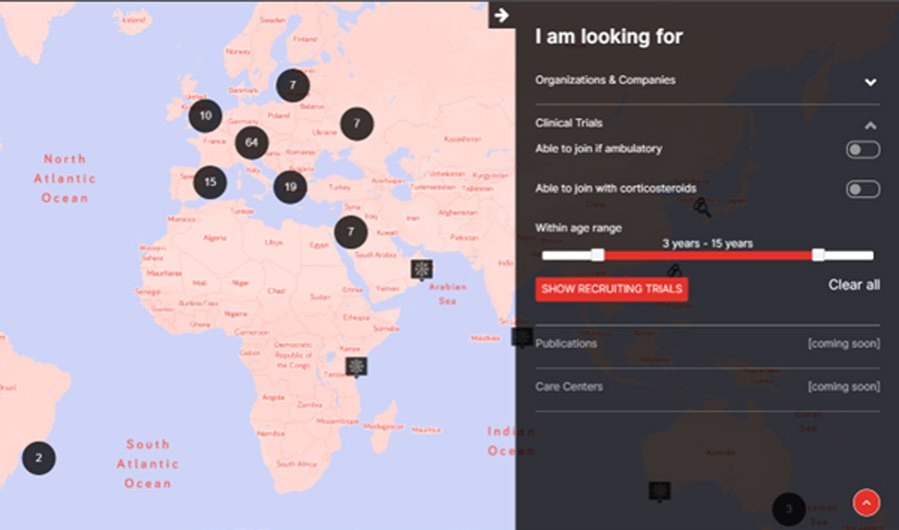
Clinical trial filter function

**A FAIR supported and secure tool:** Duchenne Map is supported by a FAIR-by-design Repository [3]. The Duchenne Data Repository can host a range of dystrophinopathy data from pre-clinical data to natural history data, clinical trial data, and patient-generated health data. Duchenne Map uses the Duchenne Data Repository to safely store and pull the data displayed. The Duchenne Map is compliant to all GDPR, security, data protection and privacy standards and policies in effect.

**Future plans:** Registration campaigns are ongoing to spread awareness of the functionality of the Duchenne Map. The filter function, as well as the user experience in Duchenne Map is continuously updated following the recommendations and feedback of the users. Duchenne Map has the potential to be leveraged for a variety of uses, with the ultimate purpose of improving the lives of the dystrophinopathy families.

**Acknowledgements**

Duchenne Map has received support by the PTC Therapeutics Strive Awards program [4].

**References**

1. Courbier, Dimond, Bros-Facer. Share and protect our health data: an evidence-based approach to rare disease patients’ perspectives on data sharing and data protection—quantitative survey and recommendations. OJRD, 2019, 14:175
2. Duchenne Map website: [https://www.duchennemap.org/]
3. Duchenne Data Repository: [https://www.duchennedatafoundation.org/project/duchenne-data-repository/]
4. PTC Therapeutics Strive Awards Program: [https://www.ptcbio.com/our-company/grants-and-donations/strive/]

## P16

### Creation of the GARDIAN patient registry for neuronopathic Gaucher Disease Type 2 and Type 3: A collaborative approach

#### Joseph Milce^1^, Suzanne Reed^1^, Lydia Braham-Chaouche^1^, Dena Jaffe^1^, Shoshana Revel-Vilk^3^, Majdolen Istaiti^2^, Elin Haf Davies^4^, Madeline Stoodley^5,*^, Tanya Collin-Histed^5^

##### ^1^Cerner Enviza, Puteaux, France. ^2^Gaucher Unit Shaare Zedek Medical Center, Jerusalem, Israel. ^3^Gaucher Unit / Pediatric Hematology-Oncology Unit, Shaare Zedek Medical Center, Jerusalem, Israel. ^4^Aparito, Wrexham, United Kingdom. ^5^International Gaucher Alliance, Dursley, United Kingdom

*Corresponding author: Tanya@gaucheralliance.org

*Orphanet Journal of Rare Diseases* 2023: P16

**Background:** Type 2 and Type 3 Gaucher Disease (GD), a rare inherited metabolic disorder, are neuronopathic GD (nGD) and often result in infant death or progressive neurological deterioration. Current drug therapies do not cross the blood brain barrier and thus do not treat nGD.

**Objectives:** To develop a patient registry specific to nGD through collaboration between patients, caregivers, clinical experts, researchers, and industry.

**Methodology:** Led by the International Gaucher Alliance (IGA), multiple stakeholders, including patients, caregivers, clinicians, and researchers, partnered to develop a web-based platform for patients with nGD and their caregivers. Questionnaires (baseline and follow-up) were designed to capture data relevant to patients, including neuronopathic Gaucher-specific Patient Reported Outcomes (nGD-PRO) and Observer Reported Outcomes (nGD-ObsRO). Qualitative interviews with patients and caregivers ensured the use of relevant terminology. Clinicians informed the process of diagnosis confirmation.

**Results:** The Gaucher Registry for Development Innovation and Analysis of Neuronopathic Disease (GARDIAN) is a global, longitudinal, prospective patient registry with no age restrictions. Available in English, French, German, Spanish, Arabic, Japanese and Chinese, GARDIAN will capture data at baseline and every 6 months for 3 years. Data collected will include enzyme/genetic results, patient characteristics, symptoms (neurological/non-neurological), medical history, treatment, and comorbidities. Patient- and caregiver-reported outcomes include the PedsQL, PGI-S, GAD-7, PHQ-9 and an nGD-PRO and nGD-ObsRO to be validated within the registry. GARDIAN obtained institutional review board approval and was launched in Spring 2022.

**Conclusion:** The contribution of multiple stakeholder perspectives in the development of GARDIAN optimizes its value as a real-world data source. The collection of data in a systematic and standardized manner will provide a research platform for improving disease understanding, supporting patients with nGD, advancing disease management, designing safer treatments, and improving patient outcomes.

## P17

### From the French national rare disease registry (BNDMR) towards ITHACA ERN registry (ILIAD): data reusability to ease the burden of data entry

#### Arnaud Sandrin^1,*^, Céline Angin^1^, Morris Swertz^2^, Fernanda De Andrade^2^, Klea Vyshka^3,4^, Alain Verloes^3,5^

##### ^1^French National Rare Disease Registry (BNDMR)—Assistance Publique-Hôpitaux de Paris, Paris, France. ^2^University Medical Center Groningen, Dept. of Genetics, Genomics Coordination Center, Groningen, Netherlands. ^3^Assistance Publique-Hôpitaux de Paris—Université de Paris, Department of Genetics, Paris, France. ^4^CERCRID, UMR 5137, “Centre de Recherches Critiques en Droit”, Université de Lyon, Lyon, France. ^5^INSERM UMR 1141 "NeuroDiderot", Hôpital R DEBRE, Paris, France

*Corresponding author: arnaud.sandrin@aphp.fr

*Orphanet Journal of Rare Diseases* 2023: P17

**Background:** European Reference Networks (ERN) are developing registries in order to make Findable, Accessible, Reusable and Interoperable (FAIR) rare diseases (RD) common data elements (CDE [https://eu-rd-platform.jrc.ec.europa.eu/set-of-common-data-elements_en]), themselves derived from French CDE [1].

Connecting ERN registries with numerous pre-existing local registries altogether is an unprecedented interoperability effort. First, regulation requires patient re-information and inter-HCPs contracts, involving law and data protection expertise. Second, compliancy with interoperability international standards comes with highly technical information technology development. Third, managing inconsistencies, data duplication, and data source tracing is mandatory in a fully automated system.

**Materials and methods:** ERN ITHACA is developing a “meta-registry” called ILIAD, connecting 71 HCPs, databases, and biobanks across the EU for patients with dysmorphic/MCA syndromes and/or intellectual disability. The registry is built on MOLGENIS open-source software, providing flexible rich data structures, user-friendly data import and querying, and FAIR interfaces for programmatic data exchange [2,3].

In France, all RD expert centers patients are registered in one National RD Registry (BNDMR [www.bndmr.fr]). Data is collected through Electronic Health Records or via the web application BaMaRa. Interoperability between HCPs and BaMaRa is based on HL7 CDA standards. The system is developed at AP-HP [4].

Both BNDMR and ILIAD gather CDE. Data reuse is then operated as described on Fig. 1. Data is downloaded from BaMaRa as an Excel file, itself uploaded securely on the Molgenis platform where it is translated, pseudonimised, and formatted. The new file eventually integrates ILIAD.

**Fig. 1:**
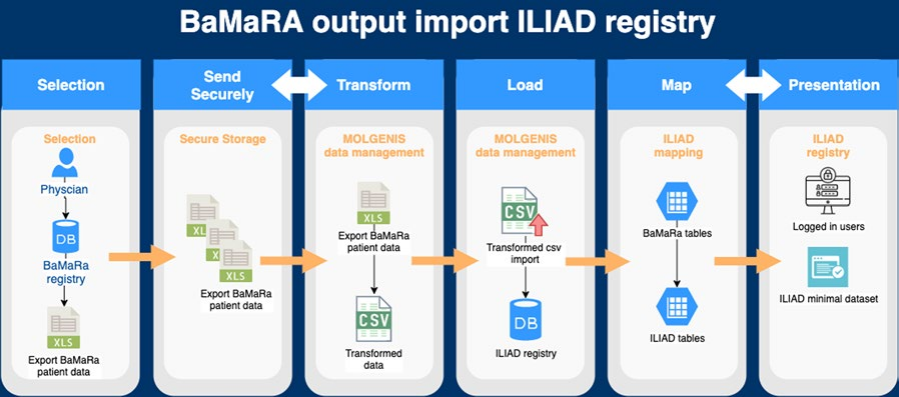
BNDMR data reuse into ILIAD

**Results:** This process allows in a simple and secure way (as double entrying data) the upload of hundreds of patients’ data by the clinician, in a few clicks and at a taylor-made frequency (every 6 months, e.g.). There is no digital interface between the two systems, minimizing cyber-risks.

Nevertheless, patients already included in BaMaRa may not make another visit at the expert center that included them. Therefore, while they are informed about BaMaRa, they have not been individually informed about ILIAD and have not yet consented to any reuse of data in ILIAD. GDPR allows alleviating information processes in some cases, e.g. if “the provision of such information proves impossible or would involve a disproportionate effort”. Discussion with local authorities should be undertaken along those lines; no conclusion is yet available.

**Conclusion:** The reusability of hundreds of patients’ data is a short-run pragmatic alternative to longer-run fully-fledged FAIR system to lift some of the data collection burden.

**References**

1. Rémy Choquet, Meriem Maaroufi, Albane de Carrara, Claude Messiaen, Emmanuel Luigi, Paul Landais. A methodology for a minimum data set for rare diseases to support national centers of excellence for healthcare and research. J Am Med Inform Assoc. 2015 Jan;22(1):76–85.
2. van der Velde KJ, Imhann F, Charbon B, Pang C, van Enckevort D, Slofstra M, Barbieri R, Alberts R, Hendriksen D, Kelpin F, de Haan M, de Boer T, Haakma S, Stroomberg C, Scholtens S, van de Geijn GJ, Festen EAM, Weersma RK, Swertz MA. MOLGENIS research: advanced bioinformatics data software for non-bioinformaticians. Bioinformatics. 2019 Mar 15;35(6):1076–1078. https://doi.org/10.1093/bioinformatics/bty742. PMID: 30165396; PMCID: PMC6419911.
3. Swertz MA, Dijkstra M, Adamusiak T, van der Velde JK, Kanterakis A, Roos ET, Lops J, Thorisson GA, Arends D, Byelas G, Muilu J, Brookes AJ, de Brock EO, Jansen RC, Parkinson H. The MOLGENIS toolkit: rapid prototyping of biosoftware at the push of a button. BMC Bioinformatics. 2010 Dec 21;11 Suppl 12(Suppl 12):S12. https://doi.org/10.1186/1471-2105-11-S12-S12. PMID: 21210979; PMCID: PMC3040526.
4. Jannot A-S., Messiaen C., Khatim A., Pichon T., Sandrin A., on behalf of the BNDMR infrastructure team. The ongoing French BaMaRa-BNDMR cohort: implementation and deployment of a nationwide information system on rare disease. Journal of the American Medical Informatics Association, Volume 29, Issue 3, March 2022, Pages 553–558

## P18

### Screen4Care: shortening diagnosis for Rare Disease Patients through Genetic Newborn Screening and Digital Technologies

#### Alessandra Ferlini^1,*^, Fernanda Fortunato^1^, Marianna Farnè^1^, Rita Selvatici^1^, Carl Rudolf Blankart^2^, Sandra Gillner^2^, Richard Roettger^3^, Jan Kirschner^4^, Joern Schenk^5^, Kaja Zarakowska^5^, Jana Zschüntzsch^6^, Michela Zuccolo^7^, Yuen Man^8^, Liz Goodman^9^, Marie Trad^10^, Stefaan Sansen^11^, Maria Martinez-Fresno^12^, Lola Tome^13^, Ludovic Baillon^14^, Edith Gross^15^, Nicolas Garnier^16,*^

##### ^1^Unit of Medical Genetics, Department of Medical Sciences, University of Ferrara, Ferrara, Italy. ^2^KPM Center for Public Management and Swiss Institute for Translational and Entrepreneurial Medicine (sitem-insel), Bern, Switzerland. ^3^Department for Mathematics and Computer Science, University of Southern Denmark, Odense, Denmark. ^4^Department of Neuropediatrics and Muscle Disorders, Medical Center, University of Freiburg, Freiburg, Germany. ^5^Takeda Pharmaceuticals International AG, Glattpark-Opfikon (Zurich), Switzerland. ^6^Universitaetsmedizin Goettingen—Georg-August-Universitaet Goettingen—Stiftung Oeffentlichen Rechts, Goettingen, Germany. ^7^F. Hoffmann-La Roche, Basel, Switzerland. ^8^Novo Nordisk Health Care AG, Zurich, Switzerland. ^9^University College Dublin, National University Of Ireland, Dublin, Ireland. ^10^Lysogene, Neuilly-sur-Seine, France. ^11^Sanofi, Chilly Mazarin, France. ^12^Illumina, Great Abington, UK. ^13^ProQR Therapeutics, Leiden, The Netherlands. ^14^PTC, South Plainfield, NJ, USA. ^15^EURORDIS, Paris, France. ^16^Pfizer Limited, Sandwich, United Kingdom

*Corresponding author: fla@unife.it, nicolas.garnier@pfizer.com

*Orphanet Journal of Rare Diseases* 2023: P18

Rare diseases (RDs) affect over 30 million people across European Union (EU) and are quality-of-life limiting or life-threatening, especially if undiagnosed and untreated [1]. Less than 10% of RD patients receive treatment and only 1% benefit from approved therapy in Europe. RD patients very often experience a “diagnostic odyssey”, enduring on average eight years without a diagnosis, leading to multiple testing, ineffective treatments, and inefficient healthcare resource utilisation. Since about 75% of RD have a genetic origin and paediatrics onset, genetic newborn screening (NBS) might represent a unique diagnostic “check point”.

Screen4Care (S4C) is a pre-competitive Public Private Partnership based on a joint effort of EU and European Federation of Pharmaceutical Industries and Associations (EFPIA). It brings together 35 partners led by the University of Ferrara and includes 21 academic partners, 9 industrial project partners (led by Pfizer), 4 small and medium-sized enterprises and EURORDIS, representing the voice of patients. The 5-year Research Project focuses on accelerating RD diagnosis through two central pillars: NBS and artificial intelligence (AI)-based tools. The project will run a pivotal genetic NBS in about 20.000 new-borns in 3 EU countries using gene panels for treatable and/or actionable RDs and offering whole genome sequencing in early symptomatic infants. New AI algorithms will be developed to identify patients beyond newborn period in Electronic Health Records (EHRs). Further, a repository of AI “symptom checkers” will be designed to facilitate suspicion and diagnosis by health care providers and patients. The S4C Virtual Clinic will complement these approaches to provide information and support post-diagnosis, with specific spaces for patients and families to meet, share experiences, network, find peer group support, as well as connect with European Reference Networks and/or to suggest referral pathways to physicians for follow-up.

S4C is organized into 6 Work Packages (WP): WP1 and WP2 are involved in digital data readiness (ethics framework and federated machine learning); WP3, WP4 and WP5 are involved in the RDs screening through genetic NBS and AI; WP6 is in charge of management, dissemination, communication and exploitation of results.

The goal of the project is to evaluate the validity of this dual approach (“genetic NBS and digital technologies”) to accelerate RDs diagnosis and improve value-based healthcare resource utilization, acceptance, and trust of families in genetic NBS, understand cost/benefit of diagnosis strategies, and their ethical considerations. First results are expected by the end of 2023.

**Acknowledgements**

*This project has received funding from the Innovative Medicines Initiative 2 Joint Undertaking (JU) under grant agreement No 101034427. The JU receives support from the European Union’s Horizon 2020 research and innovation programme* and EFPIA.

**Reference**

1. Nguengang Wakap S, Lambert DM, Olry A, et al. Estimating cumulative point prevalence of rare diseases: analysis of the Orphanet database. *Eur J Hum Genet*. 2020; 28(2):165–173

## P19

### Addressing inequalities in SMA care: a SMA European policy and access tracker

#### Adrian Harrington^1,*^, Andrea Corazza^1^, Philipp Arnold^1^, Nicole Gusset^2^, Marie-Christine Ouillade^2^, Steven Kelly^3^, Michele Pistollato^3^, Chris Jones^3^

##### ^1^Biogen, Baar, Switzerland. ^2^SMA Europe, Freiburg, Germany. ^3^Charles River Associates, Cambridge, UK

*Corresponding author: adrian.harrington@biogen.com

*Orphanet Journal of Rare Diseases* 2023: P19

Spinal muscular atrophy (SMA) is a rare, genetic, neuromuscular disease that affects individuals of all ages. It is characterized by a loss of motor neurons in the spinal cord and lower brain stem, resulting in progressive muscle atrophy and weakness. SMA presents a spectrum of disease severity. Some individuals with SMA may never sit; some sit but never walk; and some walk but may lose that ability over time. In the absence of treatment, children with the most severe form of SMA would not be expected to reach their second birthday.

While three treatments are approved in Europe, many patients are still unable to access them and the care they need, so more needs to be done to ensure no patient is left behind. To identify gaps and measure inequalities across Europe, Biogen, in collaboration with SMA Europe and Charles River Associates, conducted a comparative assessment of the policy and access landscape for SMA across 23 European countries.

Through this comparative assessment, the authors have (a) identified areas for improvement both within and across countries and (b) developed country-by-country summaries of the policy and access landscape for SMA patients. The study’s objective was to assess how different European countries are performing in several key areas, to characterise their performance with regards to the policy and access environment for SMA patients. These findings have been used to develop a consolidated set of policy recommendations that can be used to advocate for improvements at the national and European level.

Indicators include diagnosis, access pathways, access to treatment and care, political leadership and policy, and healthcare systems preparedness. The findings are illustrated in a Policy and Access Tracker and are based on publicly available information sources and country information based on a survey conducted by SMA Europe. Information was reviewed and validated by Biogen and SMA Europe national member organisations.

The SMA Tracker’s outputs consists of a White Paper presenting results and putting forward policy recommendations to tackle existing challenges, country-by-country summaries of the policy and access landscape for SMA patients and a dedicated website featuring an interactive map showing key policy and access areas that impact the life of SMA patients across the analysed countries.

In short, the ‘SMA Policy & Access Tracker’ shines a light on existing gaps affecting SMA patients and highlights actions decision-makers can take to address them.

## P20

### Orphanet Data for Rare Disease project

#### Sylvie Maiella^1^, Houda Ali^1^, Caterina Lucano^1^, Kurt Kirch^2^, Carina Thomas^2^, Stefanie Weber^2^, Marie-Cécile Gaillard^1^, Marc Hanauer^1^, C. Rodwell^1^, Ursula Unterberger^3^, Till Voigtlander^3^ and Ana Rath^1^

##### ^1^Inserm US14 – Orphanet, Paris, France. ^2^BFARM, Koln, Germany. ^3^Medical University of Vienna, Vienna, Austria

*Corresponding author: sylvie.maiella@inserm.fr

*Orphanet Journal of Rare Diseases* 2023: P20

The Orphanet Data for Rare Disease project (OD4RD) started in January 2022, it is a pilot project co-funded by the European Union’s EU4Health Programme and coordinated by INSERM. It builds on Orphanet’s specific expertise, and its organisation as a well-established network.

Orphanet is a reference network for Rare Disease (RD) information and data, with teams hosted by institutions endorsed by National Authorities. Orphanet maintains a comprehensive RD nomenclature system aligned with several non-RD specific terminology resources, allowing for semantic interoperability in a context of heterogeneous coding systems across different countries. The nomenclature is also annotated with curated information on genes, epidemiological data, phenotypic traits, and functional consequences. This unique body of knowledge, completed by textual information, is delivered in both computable and human-readable formats.

European Reference Networks (ERNs) are end-users of the Orphanet nomenclature, but also active contributors to its development, since they concentrate clinical and scientific expertise as knowledge evolves. For this reason, Orphanet’s methodology to address ERNs’ needs in terms of revision of the nomenclature has been formalised and is available online.

The project aims to fulfill the following general objectives:

- Contribute to the generation of standardised, interoperable data on RD diagnosis for primary and secondary use, through maintenance of the Orphanet nomenclature in collaboration with ERNs, and active support for its implementation in hospitals hosting ERNs.
- Contribute to the harmonisation of data collection in various settings (health records, registries) and countries, through dissemination of coding good practices at the source (health records, registries, etc.).
- Support evidence-based decision-making in the frame of the European strategy around ERNs, by providing an exploitable reference corpus of data and information on RD.

To achieve these, an Orphanet Nomenclature National Hubs Network has been set up. 13 pilot Orphanet teams participate (Austria, Belgium, Czech Republic, Germany, Finland, Spain, Italy, the Netherlands, Norway, Poland, Portugal, Sweden and Slovenia) to ensure support for local implementation of ORPHAcodes in national HealthCare Providers hosting ERNs or linked to ERNs.

Technical support is also provided to ensure the proper integration of Orphanet’s knowledge base datasets to the European Commission IT systems when needed and to provide services to the ERN community in a coordinated and user-oriented manner.

Finally, the project will produce reports on quantitative and qualitative RD coverage, overlaps and gaps amongst ERNs in order to support evidence-based decisions by the ERNs’ coordination, the Board of Member States (BoMS) and the European Commission.

## P21

### Informing without Dramas – Program to teach rare diseases to 3–18 year olds at school

#### Marta Jacinto^1,*^, Ana R. Sabino^1^, André Correia^1^, Catarina C. Duarte^1^, Paulo Gonçalves^1^

##### ^1^RD-Portugal, Lisbon, Portugal

*Corresponding author: isd.rdportugal@gmail.com

*Orphanet Journal of Rare Diseases* 2023: P21

This project aims at teaching the fundamentals of rare diseases to all students (and their teachers and families), from Preschool to Secondary School (3–18yo), being currently available from Preschool to the 9th grade. In this abstract, we will detail the project and the results of the surveys filled out by the participants on the first pilot, held for 1st to 4th grade students in December 2021. The project is free for schools, public and private.

**Method and materials:** RD-Portugal developed a set of materials: a teachers' booklet, a PowerPoint presentation and guiding notes (1st–9th grades) and a book (Preschool), and activity materials for students according to their school year.

To register, schools fill out the form available at https://raras.pt/informarsemdramatizar. Afterwards teachers attend an online training session in which they get to know these materials and the way to implement the project, and then receive the materials. Any doubts may be clarified with RD-Portugal at any time.

We have the support of ENSP (National School of Public Health) and are working with the Portuguese Directorate-General of Education (DGE).

**Status:** The project was already implemented in:

- 22 preschooler classes in 11 schools, reaching over 450 students (pilot February–June’2022);
- 98 classes from 1st to 4th grades in 23 schools, reaching over 1750 students (pilot December’2021, spread February–June’2022);
- 39 classes of 5th and 6th grades in 9 schools, reaching over 800 students (pre-pilot December’2021, pilot February–June’2022);
- 12 classes of 7th to 9th grades in 3 schools, reaching over 220 students (pre-pilot December’2021, pilot June’2022).

**Evaluation:** There are satisfaction surveys to be filled out by teachers, classes and parents. Up until now, we've analyzed those from the pilot held in 2021. For instance, in a scale from 1 (very little) to 4 (very much): teachers classified the relevance in 3.8, the adequacy in 3.5, the ease of implementation in 3.5; classes classified how much they liked the activity in 3.8. Moreover, for instance, 89% of parents stated their kids told them about the project and 77% stated their kids were able to tell them something about rare diseases.

The new classes will be filling out evaluation surveys to enable a scientific evaluation of the knowledge acquired by students.

**Conclusion:** Many schools already registered to implement the project, many more are expected to join in 2022/2023. We hope the way people deal with rare disease patients in Portugal will improve.

## P22

### The Rare and Complex Epilepsy Alliance, Italy. Promoting integration and dissemination of the European EpiCARE network through patient-led partnerships and national reference associations.

#### Isabella Brambilla^1,2,3,*^, Raffaella Rossi^1,4^, Carla Fladrowski^1,5,6^

##### ^1^Rare and Complex Epilepsy Alliance Italy, Verona, Italy. ^2^ERN EpiCARE, Department of Paediatric Clinical Epileptology, Sleep Disorders and Functional Neurology at the Hospices Civils de Lyon, Lyon, France. ^3^Dravet Italia Onlus, Verona, Italy. ^4^Famiglie Singap1 Italia, Rome, Italy. ^5^Associazione Sclerosi Tuberosa aps, Rome, Italy. ^6^European Tuberous Sclerosis Complex Association (ETSC), Wiesbaden, Germany

*Corresponding author: alleanzaepilessierare@gmail.com

*Orphanet Journal of Rare Diseases* 2023: P22

**Background:** The establishment of the European Patients Advocacy Groups (ePAGs) has enabled the representation of the European rare and complex disease communities in the European Reference Networks (ERNs). The Rare Epilepsy Alliance of Italy (REAI) was founded by EpiCARE ePAG Chair Isabella Brambilla. A nationwide network of sixteen Italian Associations (Fig. 1) in collaboration with EpiCARE Centres in Italy now advocate for patient involvement to ensure dissemination of best practice (Fig. 2).

**Fig. 1:**
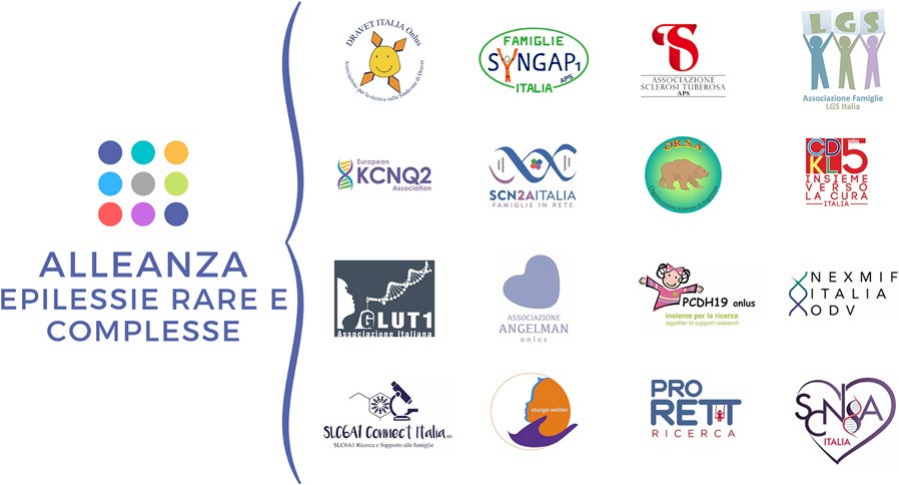
Members of Rare Epilepsy Alliance

**Fig. 2:**
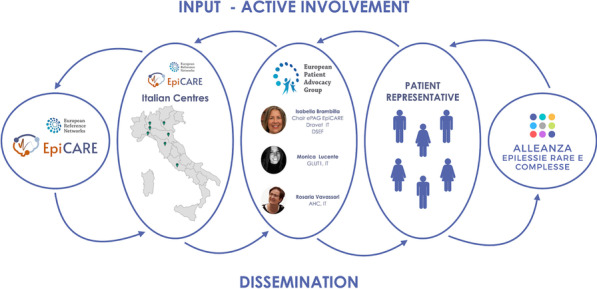
Nationwide network

**Methods:** National associations involved in rare and complex epilepsies were identified by EpiCARE ePAG Chair to establish the REAI. These associations endorsed the formation of patient-led partnerships that included the coordination of EpiCARE and its Italian Centres, thus creating a single voice for the represented pathologies.

**Results:** The REAI meets every two months and a dedicated website is now available online. The REAI has among its main objectives sharing knowledge and disseminating best practice through the development and support of an active network of Rare and Complex Epilepsy specialists (EpiCARE) and rare disease associations to encourage ePAG involvement in ERN and RD initiatives in Italy, enhance community growth and create a single voice for common and/or cross-cutting needs in considering epilepsy more than "just seizures”.

The first important goal was: the request to the Ministry for the updating of the LEAs and the recognition of all rare epilepsies, identifying with the support of a medical team, a DEE (Developmental & Epileptic Encephalopathies) macro-area/group of disease instead of proceeding by single pathologies.

The group set up an annual scientific conference with medical representatives of EpiCARE centres and patient associations (Dialog Association & ERN), that facilitate the updating of ERN-EpiCARE activities and the integration of EpiCARE projects at national level. The group identified two cross-cutting themes (Transition and drug repositioning) to set up multidisciplinary working table.

**Conclusion:** The REAI demonstrates the positive impact of patient-led involvement in ERNs. It supports a framework for integrating European activities nationally. A shift in policy towards increased collaboration between EpiCARE ERN referral clinicians and patient representatives offers a single voice for the integration of activities within the national/regional healthcare system.

**References**

1. European Reference Network for rare and complex epilepsies (EpiCARE) available at https://epi-care.eu
2. Rare and Complex Epilepsy Alliance Italy available at https://www.alleanzaepilessierare.it

## P23

### Delivering a good diagnosis: How to equip healthcare professionals to deliver an improved experience of diagnosis for people living with a rare condition

#### Natalie Frankish^1,*^, Rachel Clayton^1^, Jennifer Jones^1^, Amy Simpson^1^, Amy Hunter^1^

##### ^1^Genetic Alliance UK, London, UK

*Corresponding author: gooddiagnosis@geneticalliance.org.uk

*Orphanet Journal of Rare Diseases* 2023: P23

**Background:** The UK Rare Diseases Framework recognises the value of early diagnosis of rare conditions and identifies getting a faster diagnosis as a priority. But speed of diagnosis is only part of the picture. How a person is supported on their journey through diagnosis is equally important and all too often people with rare conditions report feeling unsatisfied [1] with their experience of diagnosis. Genetic Alliance UK set out to better understand people’s experience of diagnosis and to identify what matters most to people on their diagnosis journey.

**Materials and methods:** In November 2021, Genetic Alliance UK issued a call for people with lived experience of rare conditions to participate in the Good Diagnosis project. A total of 43 people agreed to participate and were invited to attend one of three online workshops. Each workshop invited participants to reflect on their experiences at three key stages of the diagnosis journey: the search for a diagnosis, receiving a diagnosis, and following a diagnosis. Each workshop was recorded and the discussions reviewed to identify key themes.

**Results:** The dominant themes that emerged from our workshops were:

- A person’s experience of diagnosis was significantly influenced by the healthcare professionals involved in their care.
- Clinicians who are aware and informed of rare conditions would likely be able to identify and accept the possibility of a rare condition quicker, and set in motion the referrals or tests needed to make a diagnosis faster.
- When healthcare professionals have access to reliable information and sources of support, they are better equipped to inform and support people living with rare conditions.

**Conclusions and further work:** The Good Diagnosis project confirmed that there is an urgent need to equip healthcare professionals to better support people with rare conditions.

The Good Diagnosis report [2] recommends that UK Rare Disease Framework Delivery Partners should consider developing a central repository of information on rare conditions for healthcare professionals.

This should include:

- Information on specific rare conditions
- Information on specialist services available throughout the UK including contact details and referral criteria
- Information on available support organisations
- Training materials and resources

**References**

1. Genetic Alliance UK. Rare Experience 2020: The lived experiences of people affected by genetic, rare and undiagnosed conditions. Genetic Alliance UK. 2022
2. Genetic Alliance UK. Good diagnosis: Improving the experiences of diagnosis for people with rare conditions. Genetic Alliance UK. 2020

## P24

### Collaborative approach to enhance the knowledge on paediatric rare diseases: the European Paediatric Translational Research Infrastructure

#### Lucia Ruggieri^1,*^, Adriana Ceci^1^, Bonka Georgieva^2^, Arianna Bertolani^2^, Franco Bartoloni^1^, Fedele Bonifazi^1^, Giovanni Migliaccio^2^, Annagrazia Altavilla^3^, Oscar Della Pasqua^4^, Emmanuel Mikros^5^, Hana Kubova^6^, Pierluigi Martelli^7^, Karel Allegaert^8^, Paul Dimitri^9^, Frantisek Staud^10^, Elke Smits^11^, Marina Kleanthous^12^, Sergio Giannattasio^13^, Nunzio Denora^14^, Catherine Tuleu^4^, Donato Bonifazi^2^

##### ^1^Fondazione per la Ricerca Farmacologica Gianni Benzi Onlus, Valenzano (BA), Italy. ^2^Consorzio per Valutazioni Biologiche e Farmacologiche, Bari, Italy. ^3^TEDDY Teddy European Network of Excellence for Paediatric Research / Espace Éthique PACA-Corse, AP-HM, Marseille, France. ^4^University College of London, London, UK. ^5^ATHENA Research Center, Marousi, Greece. ^6^Institute of Physiology, Academy of Sciences of the Czech Republic, Prague, Czech Republic. ^7^Alma Mater Studiorum - Università di Bologna, Bologna, Italy. ^8^Katholieke Universiteit Leuven, Leuven, Belgium. ^9^Children’s NHS Foundation Trust, Sheffield, UK. ^10^Charles University, Prague, Czech Republic. ^11^University Hospital Antwerp, Antwerp, Belgium. ^12^The Cyprus Institute of Neurology and Genetics, Nicosia, Cyprus. ^13^Consiglio Nazionale delle Ricerche – IBIOM Istituto di Biomembrane, Bioenergetica e Biotecnologie Molecolari, Bari, Italy. ^14^Dipartimento Di Farmacia - Scienze Del Farmaco, Università degli Studi di Bari, Bari, Italy

*Corresponding author: lr@benzifoundation.org

*Orphanet Journal of Rare Diseases* 2023: P24

Rare diseases (RDs) are tied hand in glove to paediatrics. 70% of genetic life-threatening RDs have an exclusively paediatric onset [1]. Collectively considered, RDs are common, as up to 36 million people in the EU live with a rare disease [https://health.ec.europa.eu/non-communicable-diseases/steering-group/rare-diseases_en]. This urges the need to increase the quality, the effectiveness, and results of research in the field.

The current work describes the contribution that a collaborative research infrastructure (RI), fully dedicated to paediatric research, could bring to the RDs field.

The European Paediatric Translational Research Infrastructure (EPTRI) has been designed as a distributed RI including research units (RUs) grouped both within Thematic Research Platforms (TRPs), according to their field of expertise, and National Nodes, according to their location.

EPTRI, under the coordination of leading scientists is able to provide centralised services managed by the Central Management Office through the Single Access Point and integrated services organised in five TRPs:

1. Paediatric Medicines Discovery TRP
2. Paediatric Biomarkers and Biosamples TRP
3. Developmental Pharmacology TRP
4. Paediatric Medicines Formulations TRP
5. Paediatric Medical Devices TRP

Such planning considered the contribution to research on new therapeutic options for paediatric RDs.

A systematic recognition of paediatric research activities and available expertise/equipment in the EPTRI paediatric RUs allowed to establish a portfolio of services that the European scientific community gathered within EPTRI could offer to stakeholders (Fig. 1). From 50 to 70% of 300 RUs included in all the TRPs deals with research on RDs, covering all therapeutic areas (rare cancers, respiratory disorders, congenic heart diseases, channelopathies, erythropoiesis disorders, mitochondrial disorders, etc.). Services offered by EPTRI in the field could support the difficult phase of translating basic and preclinical research for RDs into early clinical phases and access to treatments.

**Fig. 1:**
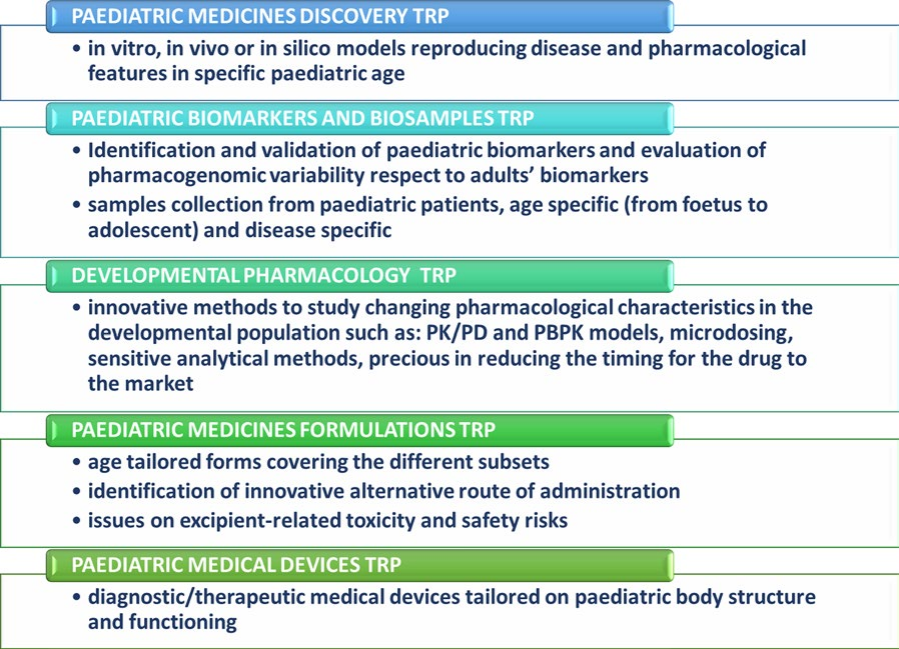
Specific contribution of EPTRI TRP to research on paediatric RDs

Particular attention has been deemed to paediatric biobanking [2], including ELSI aspects, like compliance with GDPR and assent approaches [3].

Among its activities in the field, EPTRI contributed to the organization of the 1st International Conference on Rare Diseases and Paediatric Research in November 2021 and included the themes of RDs in its Manifesto on Paediatric Research [https://www.canva.com/design/DAE4a9Pyt4w/3HspSwouiNGYQHQLTOAeNA/view].

Moreover, EPTRI is constantly promoting engagement and knowledge translation, integrating the perspectives of different stakeholders.

The consolidated and collaborative nature of the RI, its focus on paediatrics will allow researchers working together without geographical, institutional or financial barriers in a system of many interconnected research areas to bring new therapeutic options for children with RDs.

**References**

1. Nguengang Wakap S, Lambert D, Olry A, Rodwell C, Gueydan C, Lanneau V et al. Estimating cumulative point prevalence of rare diseases: analysis of the Orphanet database. European Journal of Human Genetics. 2019;28(2):165–173.
2. Catchpoole DR, Carpentieri D, Vercauteren S, Wadhwa L, Schleif W, Zhou L, Zhou J, Labib RM, Smits E, Conradie EH. Pediatric Biobanking: Kids Are Not Just Little Adults. Biopreserv Biobank. 2020 Aug;18(4):258–265.
3. Altavilla A, Giannuzzi V, Lupo M, Bonifazi D, Ceci A. Ethical, Legal and Regulatory Issues of Paediatric Translational Research. Call for an Adequate Model of Governance. Eur J Health Law. 2020 May 18;27(3):213–231.

## P25

### Delivering a good diagnosis: developing a rare conditions charter for diagnosis

#### Natalie Frankish^1,*^, Rachel Clayton^1^, Jennifer Jones^1^, Amy Simpson^1^, Amy Hunter^1^

##### ^1^Genetic Alliance UK, London, UK

*Corresponding author: gooddiagnosis@geneticalliance.org.uk

*Orphanet Journal of Rare Diseases* 2023: P25

**Background:** Genetic Alliance UK set out to better understand people's experience of diagnosis and to identify what matters most to people on their diagnosis journey. Through a series of workshops for people living with rare conditions, carers and support organisation representatives, the Good Diagnosis report identified eight guiding principles (Fig. 1) for good diagnosis. These principles capture that:

- When starting the journey to diagnosis, there is often little information and guidance on what to expect
- People with rare and undiagnosed conditions explain that fighting for the right care or treatment can be extremely challenging, particularly when unwell
- Individuals do not know their rights regarding diagnosis and healthcare.

**Fig. 1:**
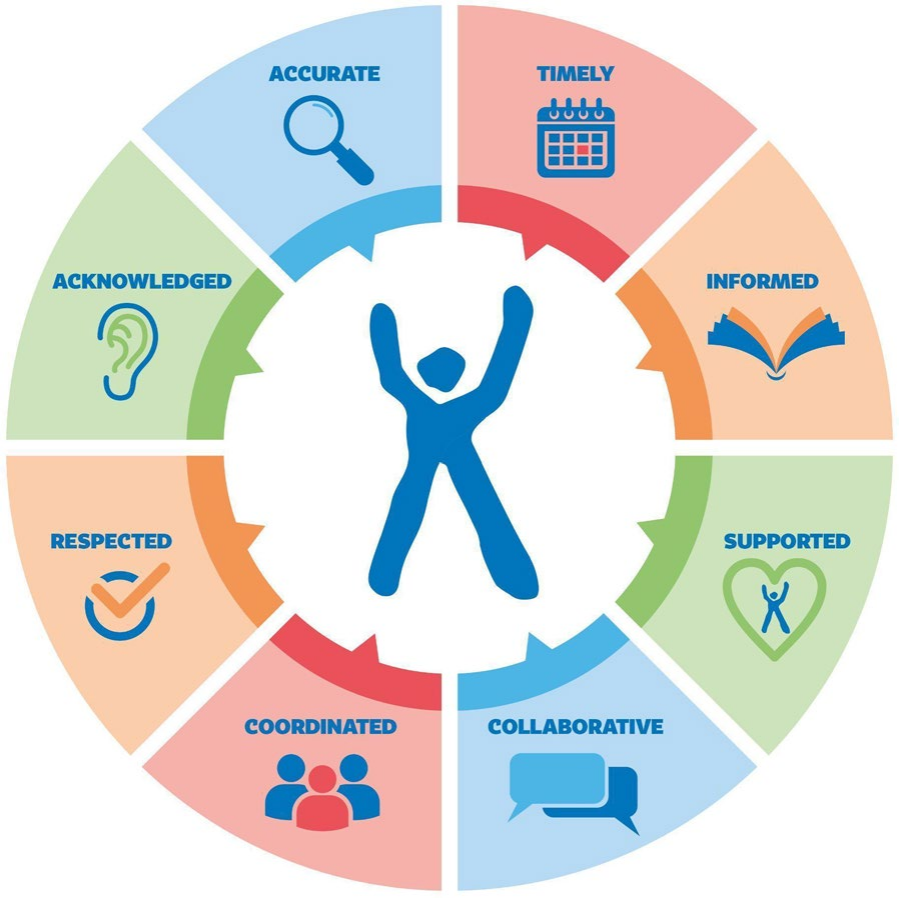
Eight principles of good diagnosis, The Good Diagnosis report

**The Good Diagnosis Patient Rights Charter:** Using the first Good Diagnosis report as the starting point, the Good Diagnosis project aims to develop a charter which allows people with rare conditions to recognise the standard of care they should expect when searching for a diagnosis and to provide them with the tools they need to enforce this.

The Charter will be based on the eight principles for diagnosis identified in the Good Diagnosis report [1].

The Charter will:

- Clearly set expectations for what care will look like during the diagnosis journey
- State how decisions about care will be made and communicated
- Provide people with details of how their information and support needs should be met
- State what can be done when a person is unsatisfied with the care they receive, and who can support them if that happens.

**Next steps:** Genetic Alliance UK will begin work on stage two of the Good Diagnosis Project, the development of a Good Diagnosis Patient Rights Charter in summer 2022. Throughout 2022, Genetic Alliance UK will work closely with people living with rare conditions, organisations that support them and health care professionals involved in their care to develop a Good Diagnosis Patient Rights Charter. This will include establishing a project steering group, developing a stakeholder survey and running consultation workshops with key stakeholder groups.

**Reference**

1. Genetic Alliance UK. Good diagnosis: improving the experiences of diagnosis for people with rare conditions. Genetic Alliance UK. 2022.

Sign up for updates on the project: https://www.smartsurvey.co.uk/s/2GBSAH/

## P26

### Coordination platform for rare diseases, a major tool to reduce inequalities overseas: the example of Guadeloupe

#### Lyne Valentino^1,*^, Dany Deschamps^1^, Maryse Etienne-Julan^1^

##### ^1^KARUKERARES—Coordination platform for rare diseases of Guadeloupe, University hospital of Guadeloupe F.W.I, Guadeloupe, France

*Corresponding author: lyne.valentino@chu-guadeloupe.fr

*Orphanet Journal of Rare Diseases* 2023: P26

As part of the third French national plan for rare diseases (PNMR3), four coordination platforms for rare diseases have been created, in four French overseas states—set up in public hospitals as one-stop offices. Purpose of the platforms is to inform and guide diagnosed and undiagnosed patients with rare disease. Designed to provide diagnostic tools and to support care courses management, platforms are patients’, health care professionals’ and social workers’ natural partners.

Coordination platform KARUKERARES is located in Guadeloupe, a French archipelago with 384.000 people living, approximately 5000 miles (8000 km) away from continental France. Between March 2021 (official start of KARUKERARES’ activities) and May 2022, 35 different cases were encountered. A longitudinal data collection already allows us to characterize our patients profiles, to analyze their requests and needs and to record platform’s responses. Thus, we observe that the majority of our patients are adult women with a rare disease diagnosed. They came to the platform by themselves, mainly searching for help to manage their care course. Besides information and listening KARUKERARES ensure patients referral to local or continental labelled rare diseases experts centers and/or towards a social accompaniment. Indeed, facilitating access to a wide structured and specific care offer (among 500 expert centers) and fostering socio-medical support, KARUKERARES address 2 local specific issues: a restricted rare diseases medical expertise and an enhanced risk of medical wandering correlated to potential loss of chance for patients and families. Offering new perspectives to improve rare diseases diagnostic and management, the « French coordination platform model» could turn out as an interesting approach for other outermost territories in Europe and also in the Caribbean—this last area counting many outermost territories.

In the future, KARUKERARES plans a triple axis road map with sensitization and training actions (notably towards GPs), e-health (e-consult and e-expertise) development and conception of a participative research program with patients and patients organization in order to better understand care courses and promote specific policies for local management of people leaving with rare diseases in Guadeloupe.

## P27

### The IGPrare European study: diversity as a source of inspiration to improve genetic information disclosure to family

#### Marion Gottrau^1^, Annagrazia Altavilla^2^, Sandra Courbier^3^, François Faurisson^4^, Roseline Favresse^3^, Anja Helm^3^, François Houyez^3^, Marion Mathieu^1,4,*^

##### ^1^Aix-Marseille University, EFS, CNRS, ADES, Marseille, France. ^2^ERER-PACA Corse, Marseille, France. ^3^Eurordis, Paris, France. ^4^Tous Chercheurs association, Marseille, France

*Corresponding author: marion.mathieu@touschercheurs.fr

*Orphanet Journal of Rare Diseases* 2023: P27

**Background:** Patients diagnosed with rare genetic diseases are often considered a moral or sometimes legal duty to inform family members of their condition. Genetic information disclosure to family members (GID) allowsan early access to genetic screening, prevention or treatments. However, GID is a tough task for patients, who might face difficulties while being fragilized by their recent diagnosis. This situation raises complex medical, ethical and legal issues. In response, European countries have adopted different approaches depending on their culture, medical practice, healthcare system and legal framework.

**Material and methods:** To address the difficulties reported by patients, we launched the *IGPrare project* in July 2020. This collaborative research aims to investigate the GID procedure and propose realistic solutions to improve its efficiency and acceptability. This project includes both French and European scales studies (IGPrare website [http://www.igprare.fr]). Here, we focused on the European study, which objective is to identify the different GID procedures across Europe. We partnered with *Eurordis* to elaborate two questionnaires disseminated during spring 2022: First one, regarding the real-life conditions, was filled during interviews with 34 patients’ associations’ representatives for 10 genetic diseases, from 16 European countries. Second one intended for geneticists and genetic counsellors, regarded the current legal framework and guidelines. It was filled by 24 respondents from 13 countries.

**Results:** (i) GID is a shared concern in Europe (Fig. 1: Patient’s associations responses to “Is GID discussed in your association?”). (ii) Regarding who is in charge of GID, associations described a high variability in real-life situations (Fig. 2), when professionals reported inter and intra-country discrepancies in legal framework and guidelines. (iii) As reported by associations, GID appears to be made in the same extent to close and “at risk” relatives (Fig. 3), although only the latter correspond to the recommended targets. (iv) Almost all associations stated the importance of healthcare professionals’ support, especially psychologists.

**Fig. 1:**
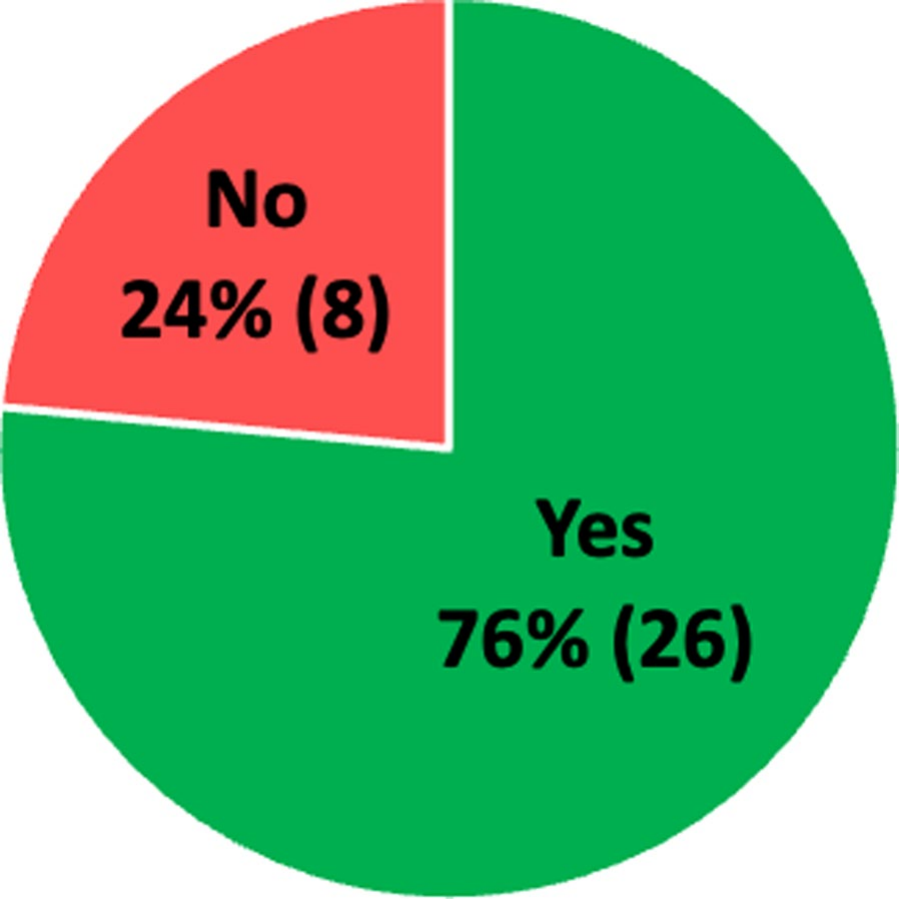
Patient’s associations responses to “Is GID discussed in your association?”

**Fig. 2:**
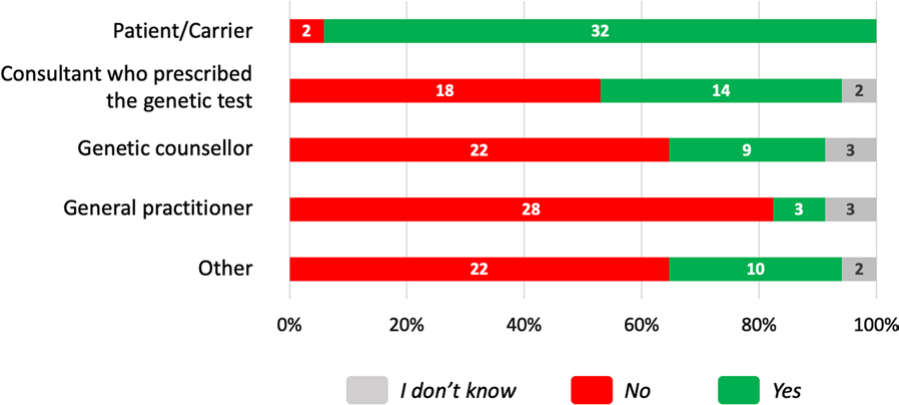
Persons in charge of GID, according to the associations

**Fig. 3:**
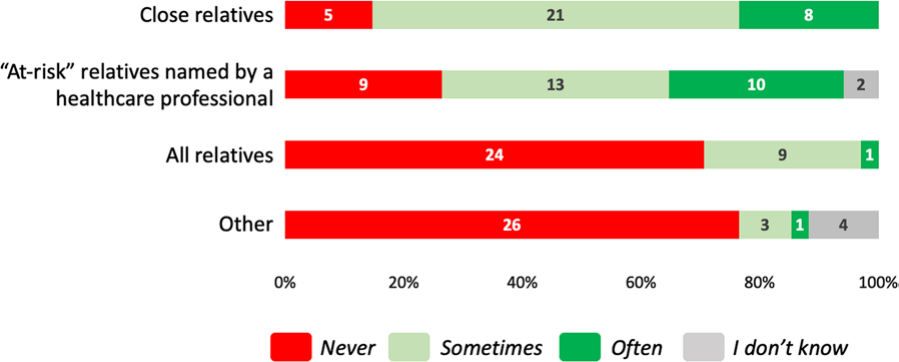
Informed relatives, according to the associations

**Conclusions:** This study enlightened the diversity of patients’ experiences in GID. Patients from different countries seem to face similar difficulties, as well as specific difficulties due to a national or cultural context. There also seem to have discrepancies between laws and guidelines framing GID to family, and its real-life conditions.

These findings will be confronted to the French scale study. The final objective is to identify and share the most efficient and acceptable solutions to improve practices in Europe, in order to facilitate genetic information disclosure to family in the future.

**Acknowledgements**

This project is supported by the French Agency of the Biomedecine (Call for proposals Research 2020, agreement 20AMP014).

## P28

### Using a patient-centric approach to understand the impact of fibrodysplasia ossificans progressiva (FOP) in an international burden of illness survey

#### Mona Al Mukaddam^1^, Katherine S. Toder^1^, Michelle Davis^2^, Amanda Cali^3,4,5^, Moira Liljesthröm^6^, Suzanne Hollywood^2^, Kim Croskery^7^, Anne-Sophie Grandoulier^8^, Elaine A Böing^9^, Jan Swiderski^8,*^, John D. Whalen^7^, Frederick S. Kaplan^1^

##### ^1^Departments of Orthopaedic Surgery and Medicine, The Center for Research in FOP and Related Disorders, Perelman School of Medicine, University of Pennsylvania, Philadelphia, PA, USA. ^2^International FOP Association, Kansas City, MO, USA. ^3^The Radiant Hope Foundation, Mountain Lakes, NJ, USA. ^4^The Ian Cali FOP Research Fund, PENN Medicine, The Center for Research in FOP and Related Disorders, Philadelphia, PA, USA. ^5^Tin Soldiers: Global Patient Identification Program, Johannesburg, South Africa. ^6^President of Fundación FOP, Argentina; Argentine Representative to the International President’s Council of the International Fibrodysplasia Ossificans Progressiva Association (IFOPA), Buenos Aires, Argentina. ^7^Ipsen, Slough, UK. ^8^Ipsen, Paris, France. ^9^Ipsen, Cambridge, MA, USA

*Corresponding author: jan.swiderski@ipsen.com

*Orphanet Journal of Rare Diseases* 2023: P28

Fibrodysplasia ossificans progressiva (FOP) is an ultra-rare genetic disorder, in which heterotopic ossification causes severe, irreversible disability. Burden of illness (BoI) studies are valuable tools for understanding the multifaceted impact of rare diseases, for which data are often limited. We detail the co-creation of a BoI survey with the International FOP Association (IFOPA), community advisors, and researchers/clinicians to understand the quality of life (QoL), social, and economic impact of FOP on patients and their family members.

The BoI survey (NCT04665323) included validated assessments and bespoke questionnaires developed to measure the impact of FOP on QoL, social/emotional wellbeing, healthcare utilisation, activities of daily living, cost of care (out-of-pocket), and employment. Resources generated by the IFOPA and input from stakeholder groups were particularly valuable in creating questionnaires to understand the wide-ranging emotional/social impacts of FOP and the everyday use of living adaptations by people living with FOP.

Multistakeholder co-creation of the BoI survey facilitated inclusion of appropriate assessments to ensure the results generated can be used to enact meaningful change for individuals and their family members. During the creation of the survey, continuous communication and transparency was central to developing an efficient working relationship among stakeholder groups. Beyond the survey, collaboration has continued with the IFOPA and community advisors to share results and raise awareness of key findings using accessible language. Dissemination of the survey through the international FOP community was instrumental to reach individuals around the world. However, one challenge of patient-centric research is designing assessments that capture the experiences of the global patient community, particularly those living in underserved areas. Sharing these learnings will help guide the development of similar, patient-centric research projects for other rare diseases and emphasise the significance of incorporating the topics of greatest importance to patients and their family members.

**Funding: **Sponsored by Ipsen.

**Disclosures: **MAM: Research investigator: Clementia/Ipsen, Regeneron; Non-paid consultant: BioCryst, Blueprint Medicines, Daiichi Sankyo, Keros; Advisory board (all voluntary): IFOPA Registry Medical Advisory Board, Incyte, International Clinical Council on FOP; non-restricted educational fund from Excel and Catalyst sponsored by Ipsen; KST: Research funding from Clementia/Ipsen and Regeneron; MD: Member of the Rare Bone Disease Alliance Steering Committee, the Rare Bone Summit Steering Committee, and the Global Genes RARE Global Advocacy Leadership Council; AC: Trustee of The Radiant Hope Foundation, Trustee of the Ian Cali FOP Research Fund/Penn Medicine, Co-founder and Advisory Board member of the Tin Soldiers Patient Identification Program, Executive Producer of the Tin Soldiers documentary, Past IFOPA Chairman of the Board, Executive Associate of the International Clinical Council (ICC) on FOP (all voluntary); ML: Co-Founder and President of Fundación FOP, Argentine IPC Representative, IFOPA Research Committee member, past IFOPA Board member (all voluntary); SH: Committee member on the IFOPA LIFE Award Program, Committee member on the IFOPA Fundraising Committee; KC, JS: Employees and shareholders of Ipsen; ASG: Employee of Atlanstat, contractor for Ipsen; EAB: Employee of Ipsen; JW: Employee of Ipsen at the time of the study; FSK: Research investigator: Clementia/Ipsen, Regeneron; Advisory Board: IFOPA Registry Medical Advisory Board; Founder and Past-President of the International Clinical Council (ICC) on FOP.

In April 2019, Ipsen acquired Clementia Pharmaceuticals.

## P29

### A transition to adult life for adolescents and adults with Duchenne or Becker muscular dystrophy- a needs survey

#### Sharon Barak^1,2,*^, Shirley Ackerma-Laufer^3^, Tali Kaplan^3^, Michal Gudinski-Elysshiv^3^, Sharon Rubinstein-Shatz^3^, kineret Perry^3^

##### ^1^Department of Nursing, College of Health Sciences, Ariel University, Ariel, Israel. ^2^Department of Pediatric Rehabilitation, The Edmond and Lily Safra Children's Hospital, The Chaim Sheba Medical Center, Ramat-Gan, Israel. ^3^Little Steps Association for Children with Duchenne Muscular Dystrophy (DMD) / Becker Muscular Dystrophy (BMD), Kfar Saba, Israel

*Corresponding author: sharoni.baraki@gmail.com

*Orphanet Journal of Rare Diseases* 2023: P29

**Background:** Treatment for Duchenne and Becker muscular dystrophy (DMD and BMD, respectively) can extend life expectancy [1–3]. Prolonging life expectancy leads to new consequences in the process of transfer from childhood to adulthood [4]. The purpose of this study is to evaluate transition to adulthood needs of people with DMD/BMD.

## Materials and methods

*Participants*

People with DMD/BMD aged ≥ 16. Participants were identified from "Little Steps" organization registry.

*Outcome measures*

1. Mobility (1-full use of assistive devices to 3-no assistive devices).
2. Respiratory function (1-full invasive respiration to 4-independent berating).
3. Transition to adulthood (Transition Readiness Assessment for Young Adults with DMD; Fig. 1) [5].

**Fig. 1:**
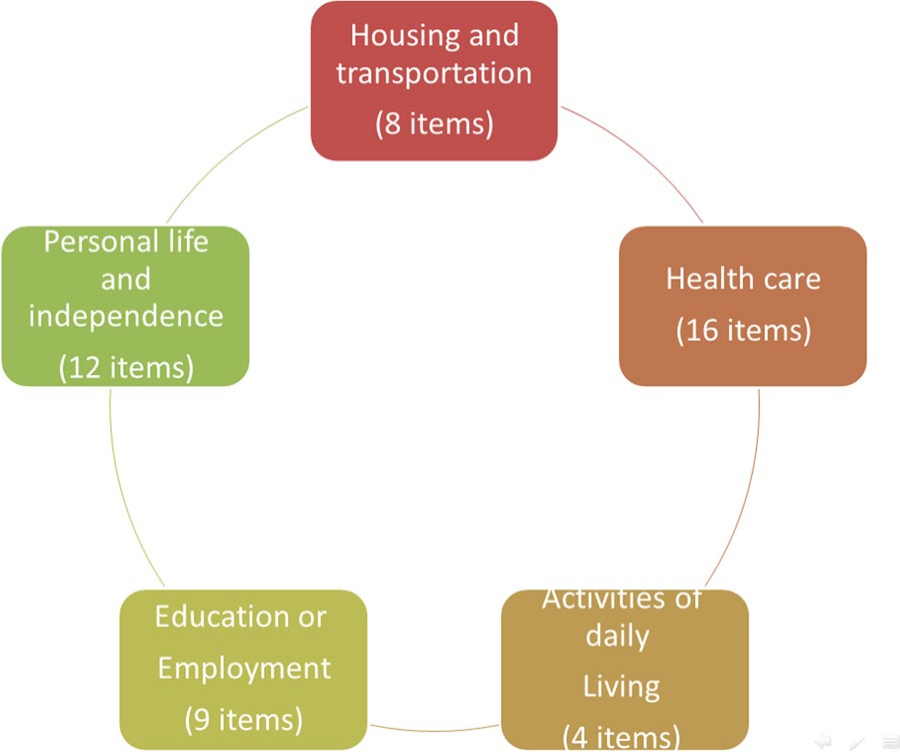
Transition Readiness Assessment for Young Adults with DMD—domains

*Data analysis*

The percentage of participants needing help was calculated. Factors associated and predicting transition were evaluated.

## Results

*Participants' characteristics*

Participated in this study 43 people with DMD/BMD (mean age: 25.8 ± 7.7; 93% males; 60.5% DMD; Table 1).

**Table 1:** Participants' characteristics

Characteristics		N (%)
Muscular dystrophy type	Duchenne	26.0 (60.5)
	Becker	17.0 (39.5)
Mobility ability	Full use of assistive devices	29.0 (67.4)
	Partial use of assistive devices	4.0 (9.3)
	No use of assistive devices	10.0 (23.3)
Respiratory functioning	Full invasive respiratory	2.0 (4.7)
	Entire day non-invasive respiratory aid	9.0 (20.9)
	Non-invasive during part tie of the day	5.0 (11.6)
	Independent	27.0 (62.8)

*Transition needs*

The transition domain in which most participants' needed help in was activities of daily living (57.7%; Fig. 2).

**Fig. 2:**
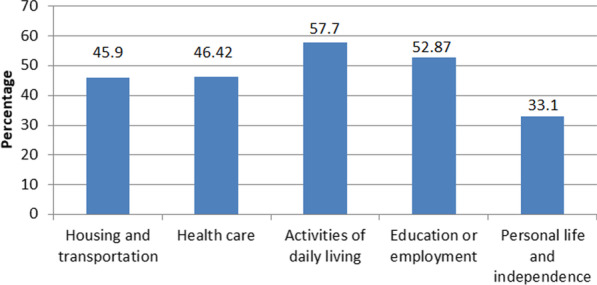
Percentage needing help in the five transition domains

Statistically significant associations were observed between most transition domains (r range: 0.35–0.94; *p* range: 0.04 to < 0.001).

*Specific transition needs*

In two items > 70% of the sample needed help: education or employment- approaching employment and education support groups (73.7%); and housing and transportation- consulting with a social worker regarding housing assistance rights (73.5%).

*Associations and prediction of transition*

Number of siblings and age positively correlated with most transition domains. Mobility ability and respiratory function did no correlate with any of the transition domains. Number of siblings predicted transition in health care (*p* = 0.03) and activities of daily living (*p* = 0.008). Older age predicted education and employment status (*p* = 0.03).

**Conclusion:** In most domains, the help needed dose not decrease with age and is not affected by function. However, adolescents and adults with more siblings require less help. There is need for care settings to support people with muscular dystrophy transition to adulthood.

**References**

1. Birnkrant et al. Diagnosis and management of Duchenne muscular dystrophy, part 1: Diagnosis, and neuromuscular, rehabilitation, endocrine, and gastrointestinal and nutritional management. *Lancet Neurol.* 2018;17:251–267.
2. Koeks et al. Clinical Outcomes in Duchenne Muscular Dystrophy: A Study of 5345 Patients from the TREAT-NMD DMD Global Database. *J. Neuromuscul. Dis.* 2017;4:293–306.
3. Birnkrant et al. Diagnosis and management of Duchenne muscular dystrophy, part 2: Respiratory, cardiac, bone health, and orthopaedic management. *Lancet Neurol.* 2018;17:347–361.
4. Rodger et al. Transition from childhood to adulthood in Duchenne muscular dystrophy (DMD) *Orphanet J. Rare Dis.* 2012;7:A8.
5. Trout et al. Transition Toolkit for Duchenne Muscular Dystrophy. *Pediatrics.* 2018;142;2:s2–s-117.

## P30

### Creating a National Programme for Rare Diseases 2019–2023: the Role of Patient Organisations

#### Risto Heikkinen^1^, Kati Saari^1,*^, Carita Åkerblom^1^

##### ^1^The Finnish Network for Rare Diseases, Helsinki, Finland

*Corresponding author: harvinaiset@harvinaiset.fi

*Orphanet Journal of Rare Diseases* 2023: P30

The rarity and high complexity of rare diseases makes it crucial to work together across various expert fields to overcome the special challenges of rare diseases. Patients’ and patient organisation’s knowledge and expertise should be recognized and promoted as a resource on all levels of the healthcare systems. In the second Finnish National Programme inclusion is one of the pivotal objectives. People living with rare diseases, their families, patient organisations and other key professionals from the field of rare diseases were involved throughout the programme, revising process to ensure the holistic view and to secure equal stand compared to people living with more common diseases.

In Finland the Ministry of Social Affairs and Health established the Sub-Committee of the Working Group on Rare Diseases, which drafted the second National Programme. The Programme was edited by a representative of the Finnish Network for Rare Diseases, Risto Heikkinen. People living with rare diseases, their families, patient organisations and other key professionals from the field of rare diseases were involved throughout the process.

1. A survey to organisations and associations representing people with rare diseases was conducted during autumn 2018.
2. A patient advocacy group (Harkko) and other member communities of the Finnish Network for Rare Diseases, consisting of non-governmental social and health organisations, were in various ways involved in evaluating the implementation of the first National Programme and in updating it.
3. A survey to the five Rare Disease Units in university hospitals and to the 14 Finnish healthcare provider members of ERN networks was conducted.
4. The national conference on rare diseases was organised in October 2018, together with round table conversations and workshops including all the stakeholders.
5. The updated draft programme was sent to the stakeholders for comments.

Measures described in the programme targeted to improve the inclusion and everyday coping of people with rare diseases, as well as their equity in access to services. Strengthening the national coordination was in key position. The Ministry of Social Affairs and Health has shown strong interest in defining measures and fostering initiatives to boost patient involvement, in cooperation with the operators in the healthcare and social welfare, including the Finnish Network for Rare Diseases, i.e., the rare disease patient organisations.

## P31

### Cross-border healthcare: Increasingly important for rare disease patients

#### Thomas Bols^1,*^

##### ^1^PTC Therapeutics, Zug, Switzerland

*Corresponding author: tbols@ptcbio.com

*Orphanet Journal of Rare Diseases* 2023: P31

Cross border healthcare is of growing importance to people with rare diseases. As new, innovative gene therapies become available, patients can be required to travel across borders, between countries in the EU/EAA/Switzerland. This can be because therapies require complex manufacture restricting how easily the therapy is transported, need highly specialised therapy delivery (e.g., through complex surgery) or because extremely low patient numbers mean companies find it difficult to launch in all countries within the current regulatory system.

Patients have the right to request cross-border care in the EU and to be reimbursed for all or part of its cost. The European framework for cross-border healthcare has two pathways:

- Directive 2011/24/EU [1]
- S2 Social Security Regulations 883/2004 and 987/2009 [2,3].

These pathways allow patients in the European Union (EU) or European Economic Area (EEA, which is made up of Iceland, Lichtenstein and Norway)), including those with rare diseases, to access medical diagnosis, treatment and prescription in any EU/EEA country and Switzerland.

The cross-border healthcare system works well for common treatments for short illnesses, or for routine procedures, such as hip replacements. The different pathways are not practical for patients with rare diseases, due to the cost of specialist treatment. Also, they are complex and less well known to patients, physicians and healthcare providers and accessing care using this pathway can be a challenging and lengthy process with no guaranteed outcome [4].

Within this context, PTC Therapeutics conducted a literature review, assessing the EU legislative and national frameworks, including relevant EU legislation and publications, and asked clarifying questions to a select number of national authorities and regulatory bodies (e.g. National Contact Points), including in Belgium, Ireland and the Netherlands. Based on this research, and in consultation with patient group representatives and healthcare professionals, PTC has developed a step-by-step guide to accessing treatment through existing cross border pathways, focusing on the S2 social security pathway. The user guide sets out to explain, in simple terms, what and how the S2 pathway works, and by, comparing national rules bring together the common features of national systems. The cross-border healthcare system should be fit for use for all people and this guide is a contribution to ensuring that access to high quality and safe care is facilitated for people living with rare diseases in Europe.

For more information on the CBHC Guide, please contact Thomas Bols at tbols@ptcbio.com.

**References**

1. Directive 2011/24/EU of the European Parliament and of the Council of 9 March 2011 on the application of patients’ rights in cross-border healthcare
2. Regulation (EC) No 883/2004 of the European Parliament and of the Council of 29 April 2004 on the coordination of social security systems (Text with relevance for the EEA and for Switzerland)
3. Regulation (EC) No 883/2004 of the European Parliament and of the Council of 29 April 2004 on the coordination of social security systems (Text with relevance for the EEA and for Switzerland)
4. Regulation (EC) No 883/2004 of the European Parliament and of the Council of 29 April 2004 on the coordination of social security systems (Text with relevance for the EEA and for Switzerland)

## P32

### Psychotherapist-facilitated group support for rare condition ‘community leaders’ established during the Covid-19 pandemic: feedback from participants and future plans

#### Amy Hunter^1,*^, Kym Winter^2^, Zubyda Azzam^2^, Lauren Roberts^1^, Isabel Rundle^1^, Amy Simpson^1^, Zaynab Nazar^3^, Joe Keenan^3^

##### ^1^Genetic Alliance UK, London, UK. ^2^RaremindsCIC, Hertford, UK. ^3^Dept of Psychology, Manchester Metropolitan University, Manchester, UK

*Corresponding author: amy.hunter@geneticalliance.org.uk

*Orphanet Journal of Rare Diseases* 2023: P32

**Background:** We developed a programme of psychotherapist-facilitated group sessions to support community leaders/patient advocates, in response to the emotional pressures of the Covid-19 pandemic. Such individuals may be their organisation’s sole point of contact or have few colleagues; many are unpaid volunteers; many are caregivers or are themselves affected by a rare condition. Although their own experience can provide high levels of insight into their community’s challenges, it may also increase the risk of burnout and vicarious trauma when their community’s need for support intensifies.

The programme had two primary aims. Firstly, to provide a safe, professionally facilitated, reflective micro-community to learn with, and from, colleagues from other organisations. Secondly, to introduce relational psychodynamic-systems thinking to help participants to:

- build emotional resilience/protect their own emotional wellbeing
- understand how to manage difficult situations when supporting their members
- develop effective boundaries between personal and professional lives.

**Methods:** Participants were recruited via Genetic Alliance UK networks. Groups were limited to ten and were facilitated by a psychodynamic psychotherapist from rareminds. Initially, two groups ran via zoom for 75 min weekly, for 12 weeks. Groups were closed to new members after week two.

Informal feedback from participants was sought by email and online survey. A formal assessment by Manchester Metropolitan University is underway consisting of semi-structured interviews which are being analysed by interpretative phenomenological analysis (IPA).

**Results:** Informal feedback was extremely positive (see example quotes below) and led to the delivery of further sessions for new participants.

*‘We’re caregivers to everyone but ourselves… this is something the facilitator really brought home all the time, that where were you in this?’*

*‘This was a life changing experience for me for the positive and just what I needed.’*

To date three participants from the most recent groups have consented to participate in the interview study. Two overarching themes have emerged: the psychosocial impact of working within the rare condition community (and key factors that can mitigate the impact), and developing resilience (a process that takes time and support, and which the group support successfully initiated).

**Conclusion:** Given the positive impact of the group work we hope to secure funding for further provision, and we encourage ‘graduates’ of the scheme to continue meeting in their groups for peer support, with monthly facilitation from a psychotherapist.

## P33

### Improvement of screening, diagnosis, and therapeutic management of patients with porphyrias in the South-West of France by the deployment of the “*Réseau Porphyries Nouvelle-Aquitaine*” (RPNA France) within the framework of the French national rare disease network

#### Mercié Patrick^1,*^, Gensous Noémie^2^, Kim Ly^3^, Roblot Pascal^4^, Fauchais Anne-Laure^3^, Martin Mickael^4^, Sturtz Franck^3^, Hauet Tierry^4^, Mattuizi Aurélien^5^, Ged Cécile^6^, Blouin Jean-Marc^6^, Le Moal Sylvie^7^, Duffau Pierre^2^, Dufois Olivier^8^, Machelart Irène^9^, Delbrel Xavier^10^, Charlanne Hilaire^9^, Vial Guillaume^2^, Wendy Jourde^2^, Ribeiro Eammanuel^2^, Morice-Picard Fanny^11^, Labrèze Christine^11^, Gouya Laurent^12^, Bubien Yann^13^ et Richard Emmanuel^6^*pour le Réseau Porphyries Nouvelle-Aquitaine (RPNA-France)*

##### ^1^service de médecine interne et immunologie clinique (PEEC-EPNET), CHU Bordeaux, INSERM 1312 BRIC BioGO, Univ. Bordeaux, France. ^2^service de médecine interne et immunologie clinique, CHU Bordeaux, CNRS UMR 5164 Immunoconcept, Bordeau, France. ^3^service de médecine interne et laboratoire de biochimie, CHU Limoges; ^4^service de médecine interne et laboratoire de biochimie, CHU Poitiers, Poitiers, France. ^5^service de gynécologie, CHU Bordeaux, ^6^laboratoire de biochimie, CHU Bordeaux, INSERM 1312 BRIC BioGO Univ. Bordeaux, Bordeaux, France. ^7^Association Française des Malades Atteints de Porphyries (AFMAP). ^8^service de médecine, CHG Sud-Gironde, Langon, France. ^9^service de médecine interne, CHG Côte-Basque, France. ^10^service de médecine interne, CHG Pau, Pau, Bayonne, France. ^11^service de dermatologie, CHU Bordeaux, CNR MAGEC-Sud, Bordeaux, France. ^12^Centre Français des Porphyries, AP-HP, Paris, France. ^13^Directeur général du CHU de Bordeaux, coordonnateur du GCS-NOVA, Bordeaux, France

*Corresponding author: Patrick.mercie@chu-bordeaux.fr

*Orphanet Journal of Rare Diseases* 2023: P33

**Background:** Porphyrias are a set of 9 diseases of hepatic or erythropoietic origin, complex and very rare with low and variable prevalence depending on the type of porphyria (from 1/25,000 to 1/106).

**Methodology:** In collaboration with the CNMR porphyria from Paris, we have created a network for the screening, diagnosis and care of patients with porphyria in the Nouvelle-Aquitaine region (RPNA) in the South-West of France (84,036 km^2^ and near of 5 million inhabitants) with the help of the French Association of Patients with Porphyrias (AFMAP) and the New-Aquitaine Health Cooperation Group (GCS-NOVA) bringing together the University Hospitals of Bordeaux, Limoges and Poitiers and their respective regional hospitals (Fig. 1). We have developed several skills: a national and European reference biochemistry laboratory (2021/2012); an expert clinical center (2020) at the Bordeaux University Hospital; various training courses (medical students and interns, general practitioners), a regional network with the collaboration of different departments of different medical specialties, multidisciplinary consultation meetings, implementation of a therapeutic education program (2022), collaboration with French gynecologists, clinical research protocols, as well as basic research work within the framework of the INSERM 1312 BRIC unit.

**Fig. 1:**
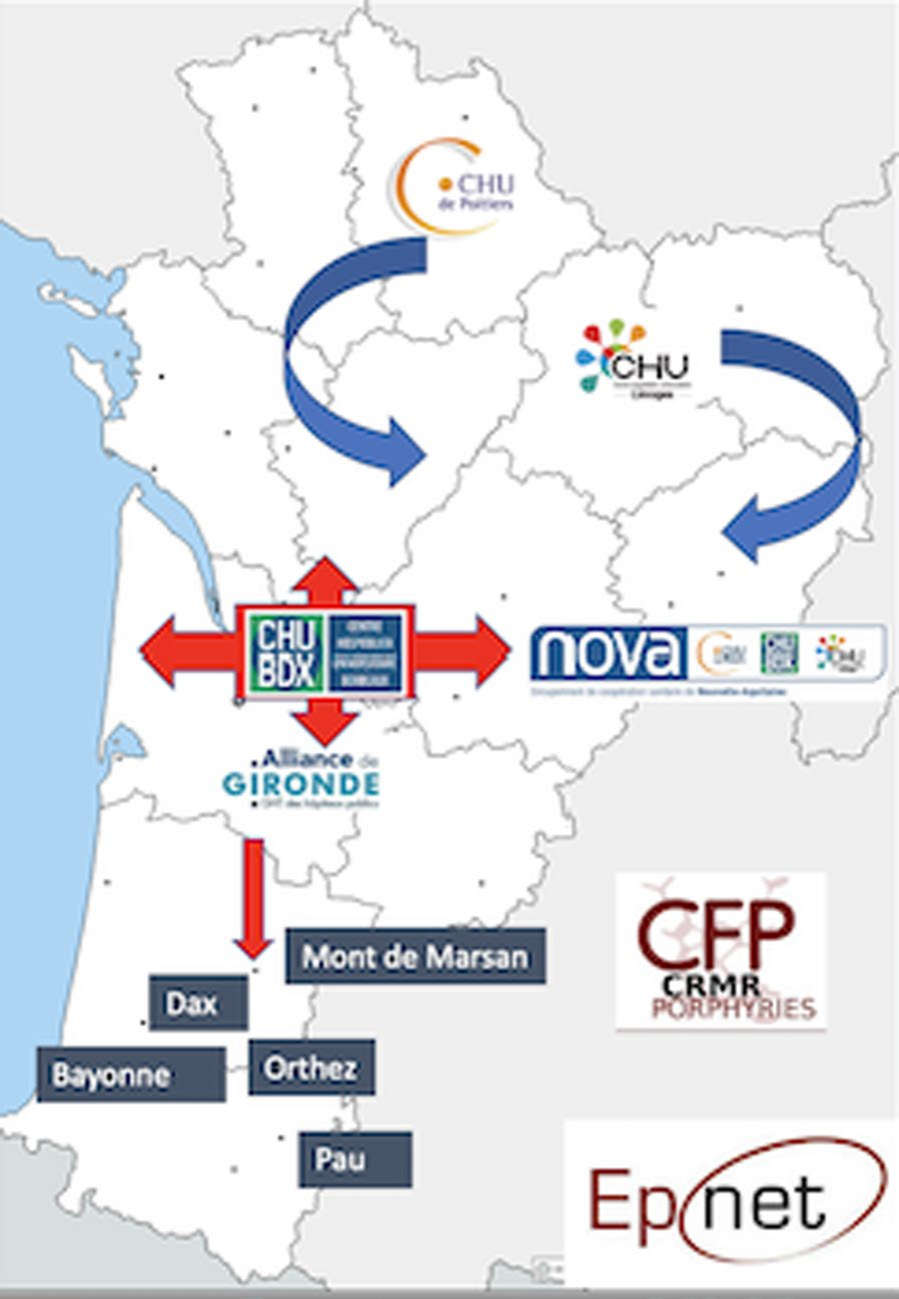
Representative map of the organization of the porphyria screening and care network in the “Nouvelle-Aquitaine” region (France)

**Discussion:** In 2 years, our active file of patients with porphyrias has increased from 25 to more than 85 patients. This has enabled close clinical and biological care of patients, face-to-face consultations and more frequent teleconsultations, the continuation of genetic investigations within affected families, more frequent screening in the general population and an optimal care offers on the territory of New Aquitaine allowing an obvious reduction of the inequality of care on the territory.

## P34

### Benefits that patients get from Rare Diseases (RD) associations, compared with inhibitors that they experience before the initial contact

#### Yvan Lattenist^1,*^

##### ^1^Rare Disorders Belgium, Erpent, Belgique

*Corresponding author: rdbelgium@yahoo.com

*Orphanet Journal of Rare Diseases* 2023: P34

It is generally accepted that 5–7% of the population is suffering from RD, but there is a clear discrepancy between these statistics and the number of contacts that RD associations can establish, whatever their efforts are to communicate: only some hundreds of people do get in touch each year while they should be tens of thousands. A paradox!

A major question for organizations caring about RD is to understand why these people are « hiding». Common reasons are known: lack of awareness about the existence of those organizations and their effective support; fear of taboo; discrimination because of lack of confidentiality versus employers, insurances, future partners…

Those common explanations are insufficient to build efficient communication on our subject matter: we need better « tools for action» for real marketing in the RD world!

As far as we know, figures have never been produced about benefits gained from interacting with RD associations in comparison with inhibitors that might induce hesitation before the first contact.

Rare Disorders Belgium has therefore organized an investigation through a survey, in June 2021. A large panel of people concerned by the topic did put evidence on various explanations which are of interest for all RD associations wanting to enhance their communication: « what are the recognized fears and inhibitors that patients face before getting in touch, compared to the benefits that they get from passing those thresholds?».

Let us, in this short abstract, name only the two predominant conclusions in each domain of our research:

- The most important benefit could have been expected but was never so clearly identified: 47% of responders recognize that they received information never provided by their medical contacts!
- The major inhibitor, although often suggested, is not the fear of lack of confidentiality, on the contrary! This factor, with a percentage of agreement of only 16%, is the least important of all suggested thresholds.

The results of this survey could be adapted to different groups of rare diseases, based on their impact (visible or not, genetically inherited, handicapping physically or mentally…). Hence, this survey could be a contributor to better communication for all RD organizations.

## P35

### A unique partnership in rare diseases: When a patient advocacy group connects with committed researchers

#### Stephanie Ernst^1,*^, E.J. (Joanne) Verweij^2^

##### ^1^TAPS Support, Almere, The Netherlands. ^2^Department of Obstetrics, division Fetal Therapy, Leiden University Medical Center, Leiden, The Netherlands

*Corresponding author: stephanie@tapssupport.com

*Orphanet Journal of Rare Diseases* 2023: P35

**Background:** Twin Anemia Polycythemia Sequence (TAPS) is a rare disease affecting monochorionic twins with little awareness. First named in 2006, many misconceptions exist about its treatment, outcomes, and diagnosis. For this reason, twins with TAPS still have poor survival rates and short- and long-term outcomes. Increasing awareness for diagnosing TAPS will save lives, and importantly help in understanding the long-term prognosis for these twins, as well as allowing families and medical teams make informed decisions about treatment together.

**Methods:** Stichting TAPS Support and the LUMC Fetal Therapy team work together to raise awareness, communicate research topics and patient concerns, and create open dialogues about TAPS.

The 15-member-strong team consists of a diverse team with patient representatives and medical advisors. They collaborate and support each other on all projects and work as equals.

**Results:** Three examples of projects that have emerged:

- Yearly ‘TAPS day’ and ‘Twin Run’ to create awareness
- Patient webinars are designed for patients to ask questions to researchers to learn more about the diagnosis. Medical professionals increase knowledge about patient's questions. Another benefit has been research insights. During conversations with patients, new research topics and new areas to explore have been raised. It has opened insights into how families cope with, and process their diagnosis. (Fig. 1)
- Short informational presentations on social media about different aspects of TAPS.

**Fig. 1:**
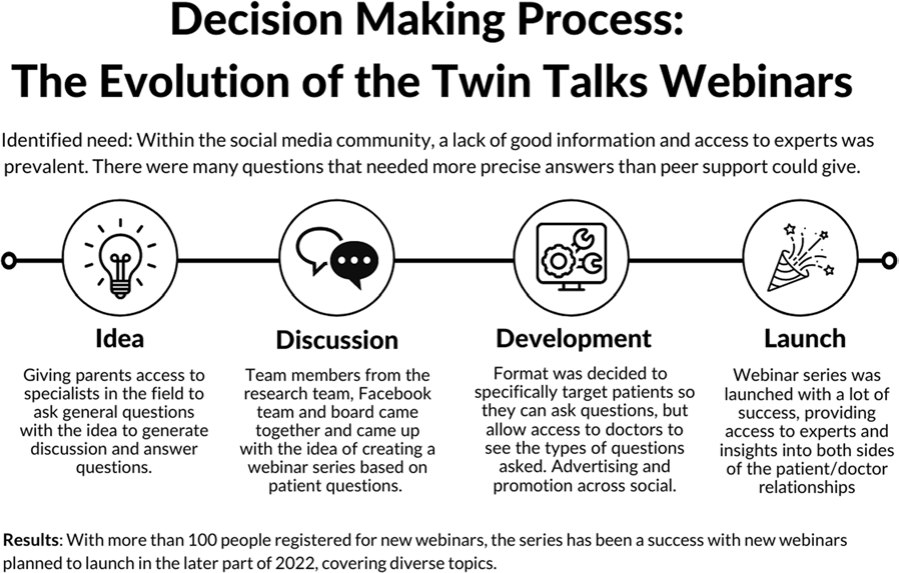
The development of the Twin Talks Webinar series, and an example of our process

**Conclusion:** For several reasons, working in collaboration with patients and researchers is essential in rare diseases.

1. Creating awareness both for patients and caregivers
2. Creating better understanding of the disease by involving patient input so new research can be initiated
3. Creating a large worldwide network of patients and caregivers for knowledge utilization

By extension, the patient group gains the reputation for giving quality information that is monitored and updated constantly; with the evolution of research. It has also created strong friendships and mutual respect between the team—each member has an equal voice.

Our key learning is to set boundaries, be open about communications, and keep everyone up to date with all information. The best approach is a sense of humor, mutual passion, respect, and the occasional shared emotional moment.

**Acknowledgements**

Stephanie Ernst writes on behalf of the TAPS support foundation, and Joanne Verweij on behalf of the LUMC Fetal Therapy team. We want to thank all involved for their ongoing collaboration.

## P36

### Innovative FAIRification solution for a Rare Disease Patient-led Registry: The Duchenne Data Platform

#### Nawel Lalout^1,4^, Mario Prieto^2^, Alberto Camara^2^, Eduardo Quemada^2^, Núria Queralt Rosinach^3^, Bruna dos Santos Vieira^4,5^, Marco Roos^3^, Peter A.C. ‘t Hoen^4^, Elizabeth Vroom^1^, Mirjam Franken^1^, Rajaram Kaliyaperumal^3^, Mark D. Wilkinson^2^

##### ^1^Duchenne Parent Project, Veenendaal, The Netherlands. ^2^FAIR Data Systems S.L., Madrid, Spain. ^3^Department of Human Genetics, Leiden University Medical Center, Leiden, The Netherlands ^4^Center for Molecular and Biomolecular Informatics, Radboud Institute for Molecular Life Sciences, Radboud University Medical Center, Nijmegen, The Netherlands. ^5^Department of Medical Imaging, Radboud Institute for Health Sciences, Radboud university medical center, Nijmegen, the Netherlands

*Orphanet Journal of Rare Diseases 2023, supp P36*

**Background:** The first Duchenne patient-led registry to undertake a FAIRification process is the Duchenne Data Platform (DDP), managed by the Duchenne Parent Project, a patient organization in the Netherlands.

The reality for patients with Duchenne and Becker Muscular Dystrophy (DMD/BMD) is that, after diagnosis, they are seen by different healthcare professionals. Consequently, a wide range of health data is produced. These are kept in different data systems and their (re)use is prevented by silo mentalities. The adoption of FAIR data practices [1] by all stakeholders who build, manage, and own these data systems can address this usage problem. As such, the DMD/BMD patient community pledged to make DMD/BMD-related data FAIR, i.e., Findable, Accessible, Interoperable, and Reusable for humans and machines.

**Methods:** The FAIRification solution called ‘FAIR-in-a-Box’ consists of an automated FAIR transformation workflow, where cloud-based servers communicate with each other through secure API calls. Variables to be collected corresponded to the Set of common data elements for Rare Diseases Registrations (CDEs) [2] and Patient Reported Outcomes (PROs). The Recommended International Standards by the European Joint Programme on Rare Diseases (EJPRD) [3] were essential to annotate, map and transform data elements into a machine-readable format. The project required a multidisciplinary team of domain and FAIR experts, supported by a dedicated FAIR project manager.

**Results:** The successful deployment of the FAIR-in-a-Box solution saw DDP reach a FAIR status within 12 months. It is innovative because:

1. It provided minimal disturbances to the source data capture and publishing system by adding a FAIR layer on top of an existing infrastructure
2. It is modular and template-based, allowing individual components to be added, removed, or synchronized with other registries, sometimes without any software changes
3. It used a generic FAIRification process, based on international standards and deployed by other registries such as VASCA Registry for Vascular Anomalies [4]

**Conclusions:** The adoption of FAIR data practices enables efficient and real-time analysis across multiple data sources. To foster change and support others with similar FAIR projects, the Duchenne Parent Project made the FAIR-in-a-Box solution open source [5]. We’re now upscaling our FAIR efforts to make DDP interoperable with other rare disease registries as well as with the European Reference Network (ERN) EURO-NMD for all rare neuromuscular diseases. Consequently, DDP FAIR data can be used, under well defined conditions, by researchers, health care providers, regulators and patients to accelerate discoveries for early diagnosis and innovative treatments.

**Acknowledgements**

We would like to express our gratitude to the FAIR experts involved directly or indirectly with the positive outcomes of our first FAIRification process. In particular to the EJPRD technical experts who contributed their time in-kind at different stages of the project. DDP FAIR project was self-funded.

**References**

1. Wilkinson MD, Dumontier M, Aalbersberg IjJ, et al. The FAIR guiding principles for scientific data management and stewardship. Scientific Data. 2016;3. https://doi.org/10.1038/sdata.2016.18
2. European Commission. Set of Common Data Elements. European Rare Disease Platform. Accessed August 16, 2022. https://eu-rd-platform.jrc.ec.europa.eu/set-of-common-data-elements_en.
3. Home · ejp-rd-vp/CDE-semantic-model Wiki · GitHub. Accessed August 16, 2022. https://github.com/ejp-rd-vp/CDE-semantic-model/wiki.
4. Groenen KHJ, Jacobsen A, Kersloot MG, et al. The de novo FAIRification process of a registry for vascular anomalies. medRxiv. 20204, 2020; 2. https://doi.org/10.1101/2020.12.12.20245951
5. van Lin-Lalout N, Roos M et al. How Patient Organizations are Driving FAIR efforts to Facilitate Research and Health Care. Journal of Neuromuscular Diseases, vol 8, no. 6, 1097–1108. https://doi.org/10.3233/JND-210721

## P37

### Launching the First Registry for Birt-Hogg-Dubé Syndrome (BHD)

#### Katie Nightingale^1,2,*^, Joshua Henderson^3^, Nina Liu^3^, Anna Webb^1,2^

##### ^1^The Myrovlytis Trust, London, UK. ^2^The BHD Foundation, London, UK. ^3^Pulse Infoframe, London, Ontario, Canada

*Corresponding author: katien@myrovlytistrust.org

*Orphanet Journal of Rare Diseases* 2023: P37

Birt-Hogg-Dubé Syndrome (BHD) is a rare complex genetic disorder that causes lung cysts, collapsed lung (pneumothoraxes), and benign skin lesions (fibrofolliculomas). About 30% of patients will develop kidney cancer and there is no cure for BHD.

The Myrovlytis Trust (https://myrovlytistrust.org) is a registered charity in England and Wales that was founded in 2007 to promote research into rare disorders, advance education of the public in medical and molecular genetics and pursue new technologies enabling treatment where there is a clear unmet clinical need such as in BHD. The Myrovlytis Trust formed the BHD Foundation (https://bhdsyndrome.org) to support, inform and empower the BHD community.

As BHD is rare, research has often involved small cohorts, stalling progress. To address this problem, the Myrovlytis Trust started working with Pulse Infoframe (www.pulseinfoframe.com) in 2022 to develop a registry platform that would allow researchers to study commonalities across the condition. One of the key goals for the project was for data to be captured in a standardized way that met with regulatory requirements so that it could be used to drive research and improve outcomes for patients and their families.

Utilising the Pulse Platform, the Myrovlytis Trust were able to deploy the registry within 2 months and the BHD Syndrome International Registry (BIRT) (https://birt.healthie.net) was launched in March 2022. In the first 3 weeks 91 people registered with the registry and some interesting insights were captured. For example, the average age of symptom onset was 35 years of age, but the average age of diagnosis is 44. This clearly shows that many people with BHD encounter the well-known rare disease diagnostic odyssey.

The speed at which BIRT was deployed is due to the collaborative approach that the Myrovlytis Trust took in relation to the project. Working closely with Pulse, they were able to bring together the BHD community and other stakeholders to launch a registry that will help drive new research into the condition.

## P38

### The OdySMA initiative—an online tool for people living with Spinal Muscular Atrophy (SMA), patient advocacy groups, and decision-makers to identify and overcome barriers regarding access to treatment and care

#### Laura Gumbert^1,*^, Yasemin Erbas^1,2,3^, Ferdinand Schmieder^1^, Nicole Gusset^1,4^

##### ^1^SMA Europe, Freiburg, Germany. ^2^Spierziekten Vlaanderen, Borgerhout, Belgium. ^3^Tilburg University, Tilburg, Belgium. ^4^SMA Schweiz, Heimberg, Switzerland

*Corresponding author: laura.gumbert@sma-europe.eu

*Orphanet Journal of Rare Diseases* 2023: P38

The term ‘OdySMA’ is derived from Homer’s epic Odyssey adventure. This initiative aims to reveal the 'quest to access' to treatment and care of people living with spinal muscular atrophy (SMA). SMA is a rare progressive neuromuscular condition. There is a wide spectrum of how severely children and adults are affected and symptoms vary from person to person. SMA may affect daily activities such as breathing, eating, hugging, grabbing, nodding, sitting and walking.

The patient community is represented by SMA Europe, a non-profit umbrella patient organization with members from across Europe. As access to medicines, treatment and care varies substantially across European countries, SMA Europe members have increasingly requested support to better understand the European situation. Access hurdles, advocacy and policy shaping initiatives to overcome them, and to ensure continuity of care in the future pose challenges to members.

Considering the urgent need for SMA Europe to picture how fragmented access to treatment and care is, SMA Europe is developing an interactive online access atlas called OdySMA. The objectives of this advocacy tool are threefold:

1. to map the situation on access to treatment and care in European countries;
2. to inform research priorities, to identify data gaps that influence access and to promote research to fill those;
3. to help understand access differences across countries and patient subgroups, identify access priorities, gain the needed advocacy skills and build successful advocacy strategies to improve access.

OdySMA is a dynamically designed tool that visualizes and centralizes systematically collected data to illustrate access pathways. To optimize the impact, we collect “real-time” data by involving stakeholders like the pharmaceutical industry as well as the SMA community to humanize data and to reflect the patient experience. We expect to have a digital access tool covering all 23 member countries which will also be the basis for advocacy trainings for our national delegates.

Our results can be used to inform patient advocates as well as international decision-makers about patients’ unmet needs. Findings can help to counteract identified access hurdles and health inequalities. Furthermore, strategies to inform patients will be promoted and their position in the healthcare ecosphere strengthened. By being the project lead, the SMA community remains not only object of research but gets empowered to stimulate targeted research to close identified data gaps. OdySMA can be a model for other patient advocacy groups.

## P39

### Supporting patients to maximise their impact in Health Technology Assessment: a pilot project

#### Josie Godfrey^1,*^, Lindsay Birrell^1^, Rick Thompson^2^, Philippa Norman^2^

##### ^1^Realise Advocacy Ltd, London, UK. ^2^Beacon For Rare Diseases, Cambridge, UK

*Corresponding author: josie@realiseadvocacy.com

*Orphanet Journal of Rare Diseases* 2023: P39

Rare disease patient advocacy group (PAG) involvement in Health Technology Assessment (HTA) can be essential to aid decision-making, especially for conditions with limited published data or significant uncertainties about disease progression, burden of illness and the suitability of clinical trial endpoints to fully capture the impact of a new treatment. While some rare disease PAGs are experienced in this area, most are not. Equitable and sustainable practical support is needed to ensure they are able to actively participate in these processes.

Realise Advocacy piloted a semi-structured support programme for rare disease PAGs that aimed to ensure participating PAGs had an achievable action plan and were better prepared for engagement in drug development and HTA processes. The pilot also aimed to test our understanding of factors affecting PAG participation and of the support needs of PAGs. The pilot was evaluated with surveys and interviews with the 6 participating PAGs.

Key factors contributing to successful PAG involvement in HTA and access processes were identified. These included:

- Having a strategy/plan that included drug development and access
- Engaging early with pharmaceutical companies, the relevant disease community and other key stakeholders such as HTA bodies.
- Staff capacity and dedicated time
- Accessing or building skills and expertise
- Financial resources to support participation

The PAGs valued independent support that was practical rather than theoretical, required a limited time commitment and was able to fit around other demands on staff/volunteers. PAGs also noted a need for ongoing support to refine and implement action plans.

The pilot included a selection process, a readiness assessment, structured workshops, action planning resources and one to one support sessions. At the end of the pilot, all PAGs had a prioritised action plan and reported increased confidence and a better understanding of HTA requirements and how to translate their experiences into evidence. They had identified additional needs around drug development and HTA processes and wanted practical support in this area. They had concerns about whether they would be able to access the financial resources to obtain support and deliver on their priorities.

Recommendations include ensuring the availability of practical support for rare disease PAGS seeking to participate in drug development and access process; providing greater clarity from key stakeholders such as NICE and the ABPI on what funding routes are acceptable for support in this area; and considering a centralised fund that could ensure sustainable and equitable support for all rare disease PAGs.

**Acknowledgements**

Funding for the project was provided by Pfizer Inc, and Alexion Pharmaceuticals Inc.

## P40

### ERN-EYE GOOD PRACTICES SERIES: Do’s and don’ts on Usher Syndrome

#### Dominique Sturz^1,*^, Isabelle Audo^2^, Caroline Wernert-Iberg^3^, Maximin Bégin^3^, Dorothée Leroux^3^, Hélène Dollfus^3^

##### ^1^BSVÖ Austrian Association for the Blind & Partially Sighted, Usher Deafblind Forum Austria, Pro Rare Austria, Wien, Austria. ^2^CHNO des XV-XX & Institut de la Vision, Paris, France. ^3^ERN-EYE Coordination Team, Hôpitaux Universitaires de Strasbourg, France

*Corresponding author: d.sturz@utanet.at

*Orphanet Journal of Rare Diseases* 2023: P40

**Background:** Based on a collaboration with a representative of an Austrian patient association for Usher Syndrome, the European Reference Network for Rare Eye Diseases (ERN-EYE) launched a leaflet (Fig. 1) aiming to guide healthcare professionals how to welcome and how to communicate with patients with Usher Syndrome at the hospital. This good-practices leaflet was created to address patients’ expectations and improve their experience in the network’s hospitals but also in all other clinical services (Fig. 2).

**Fig. 1:**
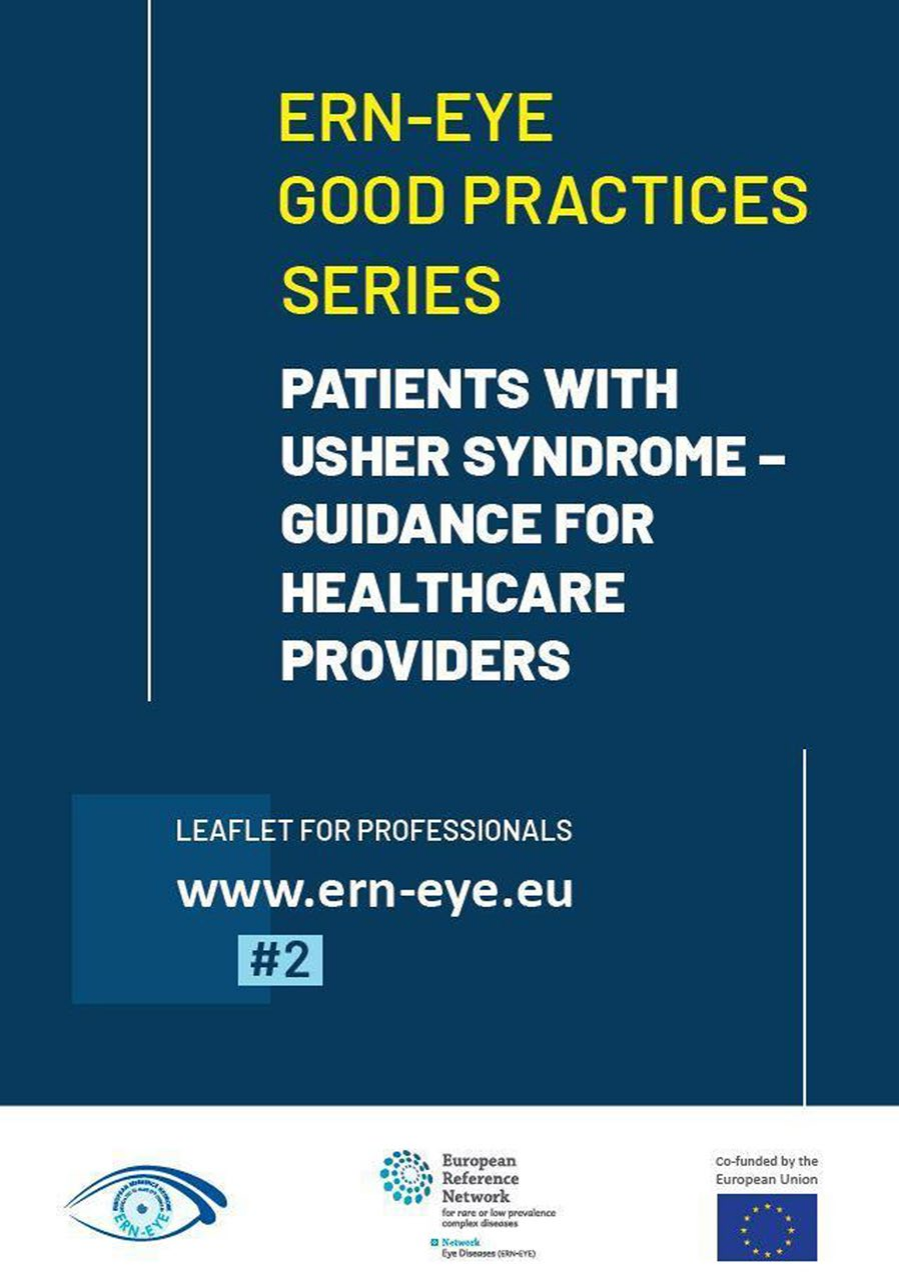
Cover of the good practices leaflet on Usher Syndrome

**Fig. 2:**
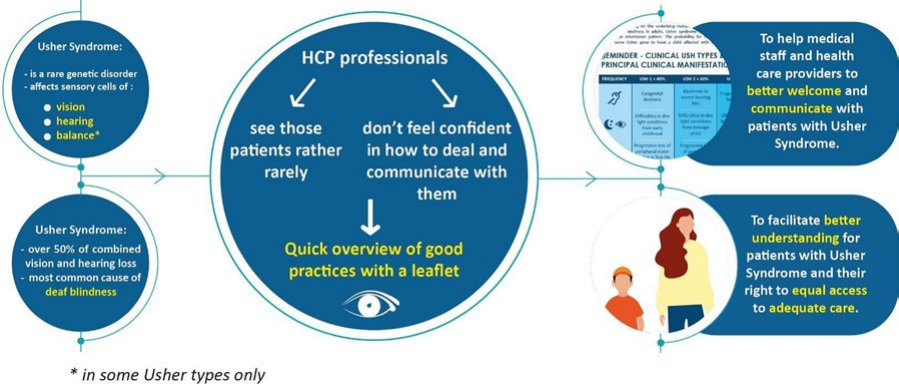
Strategic development of the leaflet: issues and objectives

**Materials and methods:** For this project, the ERN-EYE management team worked together with a representative of a patient association and health care experts of the network to gather all important information about Usher Syndrome following a structured approach (Fig. 3). The final version of the leaflet was reviewed and validated by the European Patient Advocacy Group (ePAG) representatives as well as experts of the network.

**Fig. 3:**
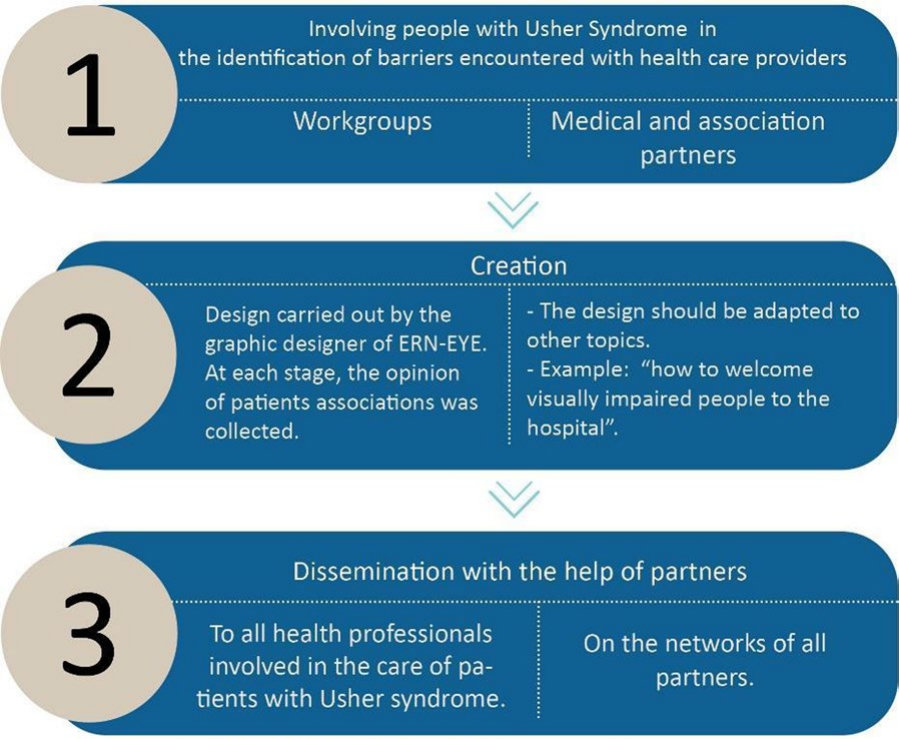
The project approach was structured around several steps

**Fig. 4:**
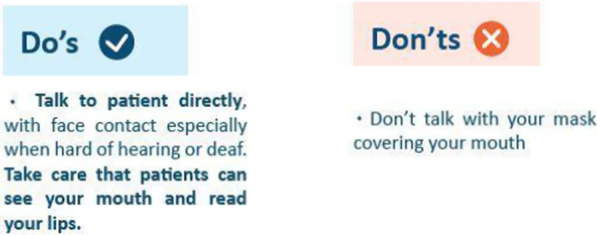
Example of different do’s and don’ts listed in the leaflet

**Results:** The leaflet is divided in several parts: clinical manifestation, identified genes, progression of the disease through the years and do’s and don’ts. The clear graphic layout makes it easy to find the essential information to retain (Fig. 4).

**Conclusions:** The leaflet will be widely distributed within the ERN-EYE network and relevant associations during internal congresses and related conferences. Thus, as it was created thanks to the involvement of patients and healthcare professionals, it fits a real need. It can be translated into several languages as required.

Finally, the project of producing a video associated with the leaflet will enhance dissemination of the information on a wider range.

**Acknowledgements**

**Fig. 25:**
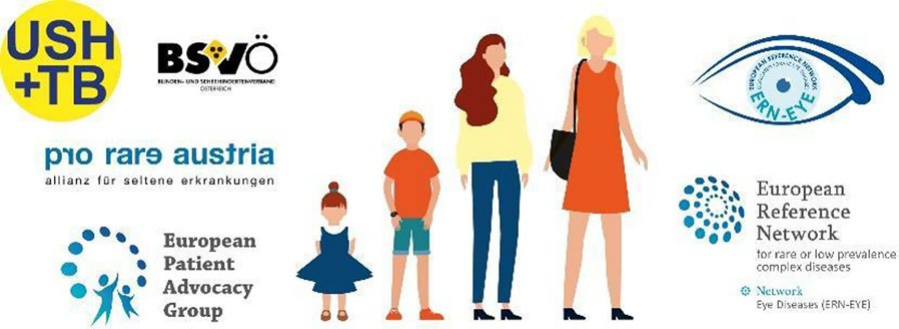
@5 All partners of the project

For this project, we wanted to involve users’ representatives to fully integrate them in the development of the system so that it reflects their needs and experiences. The partnership was obvious with the ERN-EYE European Patient Advocacy Group (ePAG) representative of an Austrian patient association for Usher Syndrome, as well as with medical experts of the network (Fig. 25 @5). The English text will go through proofreading by our English ePAG representative.

## P41

### The H-CARE project: monitoring the healthcare experience of people living with rare diseases

#### Jessie Dubief^1,*^

##### ^1^Rare Barometer Programme Senior Manager, EURORDIS-Rare Diseases Europe, Paris, France

*Corresponding author: jessie.dubief@eurordis.org

*Orphanet Journal of Rare Diseases* 2023: P41

**Aims:** The H-CARE project aims to develop a patient feedback mechanism that allows to regularly measure the clinical care experience of people living with rare diseases while ensuring robust, comparable, and independent data collection and results, across the 6000 + rare diseases and across countries.

**Background:** A pilot survey tested the feasibility of a patient feedback mechanism and of measuring rare disease patients’ and carers’ experience of clinical care. Because no Patient Reported Experience Measure (PREM) has been developed specifically for rare diseases, this pilot used the PACIC questionnaire [1] for common chronic diseases. It was disseminated from December 2019 to March 2020, to 3905 respondents in 65 countries and 23 languages, living with 900 + rare diseases. This pilot shows that rare disease patients give their clinical care experience a medium–low score (2.5 on a scale from 1 to 5) and seem to have a worse experience of health care than patients with chronic diseases [2]. It also shows that the PACIC questionnaire does not encompass all the aspects of clinical care that are specific to rare diseases, such as emotional support, genetic counselling, coordination between local care and expert centres or coordination between social and medical care.

**Method:** Based on the results of the pilot survey, the H-CARE project includes the development and validation of two PREMs, one for rare disease patients and one for family members caring for rare disease patients. A scoping literature review is being conducted that, together with upcoming focus groups, will lay the foundation for the definition of a theoretical model of high-quality experience of clinical care for rare diseases that will be based on patients’ needs. This theoretical model will then allow to design two questionnaires in several languages, before validating them.

**Discussion:** Our goal is that by 2026, the two PREMs could be used to measure the clinical care experience of people living with rare diseases as part of a regular patient feedback mechanism, and to monitor the patient centricity of the 24 European Reference Networks (ERNs) that bring together experts from across Europe to ensure that people living with rare and complex diseases can benefit from the best treatment and advice available for their condition.

**Acknowledgements**

The H-CARE project was initiated by ERN ErkNET (Franz Schaeffer, Claudia Sproedt, Vera Cornelius, Giulia Bassanese), ERN eUROGEN (Wout Feitz, Michelle Battye, Dalia Aminoff), ERN Genturis (Ignacio Blanco, Nicola Reents) and ERN LUNG (Thomas Wagner, Hilde de Keyser, Bernhard Rindlisbacher, Keerthana Iyer). It is supported by Rare Barometer, a EURORDIS-Rare Diseases Europe initiative (Sandra Courbier, Jessie Dubief, Ines Hernando, Matt Bolz-Johnson). The H-CARE Scientific Working Group also includes Prof. Dr. Michel Wensing (Heidelberg University Hospital), Prof. Dr. Elena Levtchenko (Katholieke Universiteit Leuven), Prof. Augustina Jankauskienne (Vilnius University Hospital Santaros Klinikos), Dr. Benoît Arnoult (ICON-PLC) and Dr. Samantha Anthony (SickKids). Emily White (ENDO-ERN) contributed to the scoping literature review.

**References**

1. Glasgow R.E., Wagner E.H., Schaefer J., Mahoney L.D., Reid R.J., Greene S.M. Development and validation of the Patient Assessment of Chronic Illness Care (PACIC). Medical Care. 2005, 43(5):436–44. PMID: 15838407.
2. EURORDIS, Improve our experience of healthcare! Key findings from a survey on patients’ and carers’ experience of medical care for their rare diseases, January 2021. https://download2.eurordis.org/rbv/HCARE/HCARE_FS_long.pdf

## P42

### VASCA Magazine: a tangible result from the 5-year collaboration of Patient Advocates with the clinicians of the Vascular Anomaly (VASCA) Working Group of VASCERN

#### Maria Barea^1^, Caroline van den Bosch^2^, Eulalia Baselga^3^, Laurence Boon^4^, Petra Borgards^5^, Andrea Diociaiuti^6^, Anne Dompmartin^7^, Veronika Dvorakova^8^, Maya El Hachem^6^, Paolo Gasparella^9^, Nader Ghaffarpour^10^, Emir Haxhija^9^, Alan Irvine^8^, Friedrich Kapp^11^, Kristiina Kyrklund^12^, Jochen Rößler^11,13^, Päivi Salminen^12^, Lex van der Heijden^14^, Carine van der Vleuten^15^, Aaike van Oord^16^, Leo Schultze Kool^17^, Miikka Vikkula^4,18^

##### ^1^VASCAPA, Vascular Anomaly Patient Association, Brussel, Belgium; VASCERN European Patient Advocacy Group (ePAG). ^2^HEVAS, Patient Organisation for Vascular Anomalies, The Netherlands; VASCERN European Patient Advocacy Group (ePAG). ^3^Department of Dermatology, Hospital Sant Joan de Déu, Barcelona, Spain; VASCERN VASCA European Reference Centre. ^4^Center for Vascular Anomalies, Division of Plastic Surgery, Saint-Luc University Hospital, Brussels, Belgium; VASCERN VASCA European Reference Centre. ^5^Federal Association of Congenital Vascular Malformations, Germany; VASCERN European Patient Advocacy Group (ePAG). ^6^Dermatology Unit and Genodermatosis Unit, Genetics and Rare Diseases Research Division, Bambino Gesù Children’s Hospital, Rome, Italy; VASCERN VASCA European Reference Centre. ^7^Dermatology Department, CHU Caen, University Caen Normandie, Caen, France. ^8^Paediatric Dermatology, Our Lady’s Children’s Hospital Crumlin; National Children’s Research Centre; Clinical Medicine, Trinity College Dublin, Ireland; VASCERN VASCA European Reference Centre. ^9^Department of Paediatric and Adolescent Surgery, Medical University of Graz, Graz, Austria VASCERN VASCA Associated Centre. ^10^Department of Reconstructive Plastic Surgery, Karolinska University Hospital, Stockholm, Sweden; VASCERN VASCA European Reference Centre. ^11^Division of Pediatric Hematology and Oncology, Department of Pediatrics and Adolescent Medicine, Medical Centre – University of Freiburg, Germany; VASCERN VASCA European Reference Centre. ^12^Department of Pediatric Surgery, HUS Rare Disease Center, Helsinki University Hospital and University of Helsinki, Helsinki, Finland; VASCERN VASCA European Reference Centre. ^13^Division of Pediatric Hematology and Oncology, Department of Pediatrics, Inselspital, Bern University Hospital, University of Bern, Switzerland; VASCERN VASCA Collaborating Center. ^14^CMTC-OVM (Patient Organisation Cutis Marmorata Telangiectatica Congenita and Other Vascular Malformations), the Netherlands; VASCERN European Patient Advocacy Group (ePAG). ^15^Department of Dermatology, Radboudumc Expertise Center for Haemangiomas and Congenital Vascular Malformations Nijmegen (Hecovan), Radboud University Medical Center, Nijmegen, The Netherlands; VASCERN VASCA European Reference Centre. ^16^LGD Alliance Europe (Lymphangiomatosis and Gorham's Disease), the Netherlands; VASCERN European Patient Advocacy Group (ePAG). ^17^Department of Radiology, Radboudumc Expertise Center for Haemangiomas and Congenital Vascular Malformations Nijmegen (Hecovan), Radboud University Medical Center, Nijmegen, The Netherlands; VASCERN VASCA European Reference Centre. ^18^Human Molecular Genetics, de Duve Institute, University of Louvain, Brussels, Belgium

*Orphanet Journal of Rare Diseases 2023, supp P42*

**Background:** The effective partnership between VASCERN-VASCA clinicians and patient representatives has translated in several outcomes shared at European and International level. Yet, there was no magazine for patients and care providers at European level.

**Methods:** VASCERN (European Reference Network on Rare Multisystemic Vascular Diseases) is structured in 6 working groups. The VASCA-WG (Vascular Anomalies) is composed of 14 nationally endorsed multidisciplinary Vascular Anomaly Centers and 7 ePAG (European Patient Advocacy Groups, or patient organizations’ (PO) representatives).

VASCA ePAGs and clinicians have established a strong collaborative relationship through the first 5 years or the ERN.

Representatives from the initial POs in VASCA initiated, content drafted, and managed the creation of the first VASCA Magazine.

The articles were written by VASCA (or related) clinicians, members of the POs and VASCERN’s coordination team. An external graphic designer created the layout. The project had EU-funding.

**Results:** The VASCA Magazine (cover in Fig. 1) is the first patient organization -based European magazine targeted for care providers and people affected by vascular anomalies. It aims at promoting knowledge on vascular anomalies and empowering patients.

**Fig. 1:**
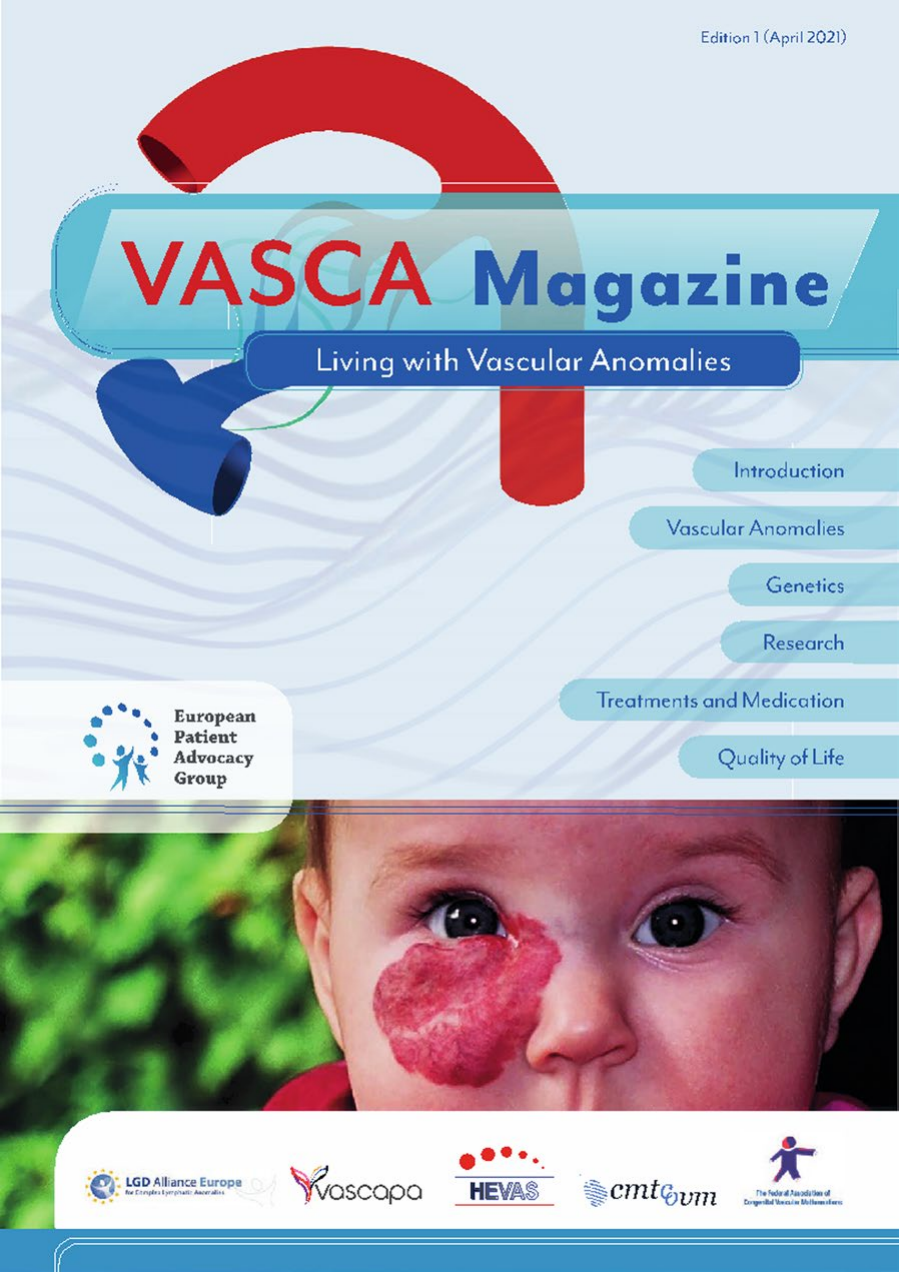
Cover of the 1st issue of the VASCA Magazine

Structured in 6 chapters (index in Fig. 2) it covers:

- State of the art scientific articles written in plain language and complemented with testimonials of affected people or caregivers.
- The work & services of VASCA patient organisations.
- What the ERNs, VASCERN, and VASCA are, the work they do to achieve better care for patients and some key projects.

**Fig. 2:**
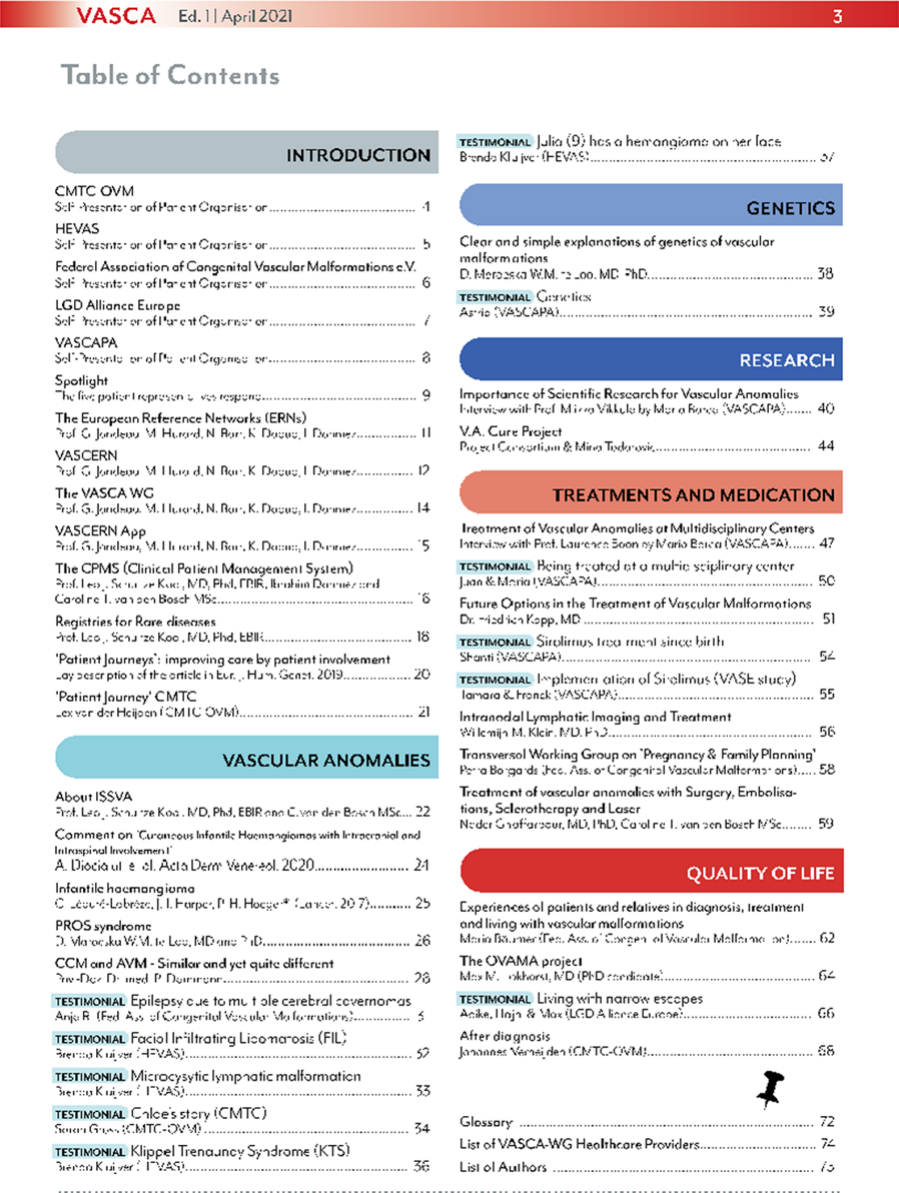
Index of the 1st VASCA Magazine

The first edition was a 72-page digital magazine published in April 2020 through VASCERN‘s and the PO‘s websites and social media. Printed copies and a flyer were also used as promotional tools.

The magazine was broadly recognized as valuable by doctors and patients. It was presented as an example of collaboration at EURORDIS’s (Rare Diseases Europe) ePAG Annual Meeting “Inspiring Collaboration and Fostering Partnership in the ERNs" (2021), at ECRD2022 (European Conference on Rare Diseases and Orphan Products) and shared at 2022 ISSVA (International Society for the Study of Vascular Anomalies) International congress.

The VASCA Magazine is available at VASCERN’s website [https://vascern.eu/expertise/rare-diseases-wgs/vasca-wg/].
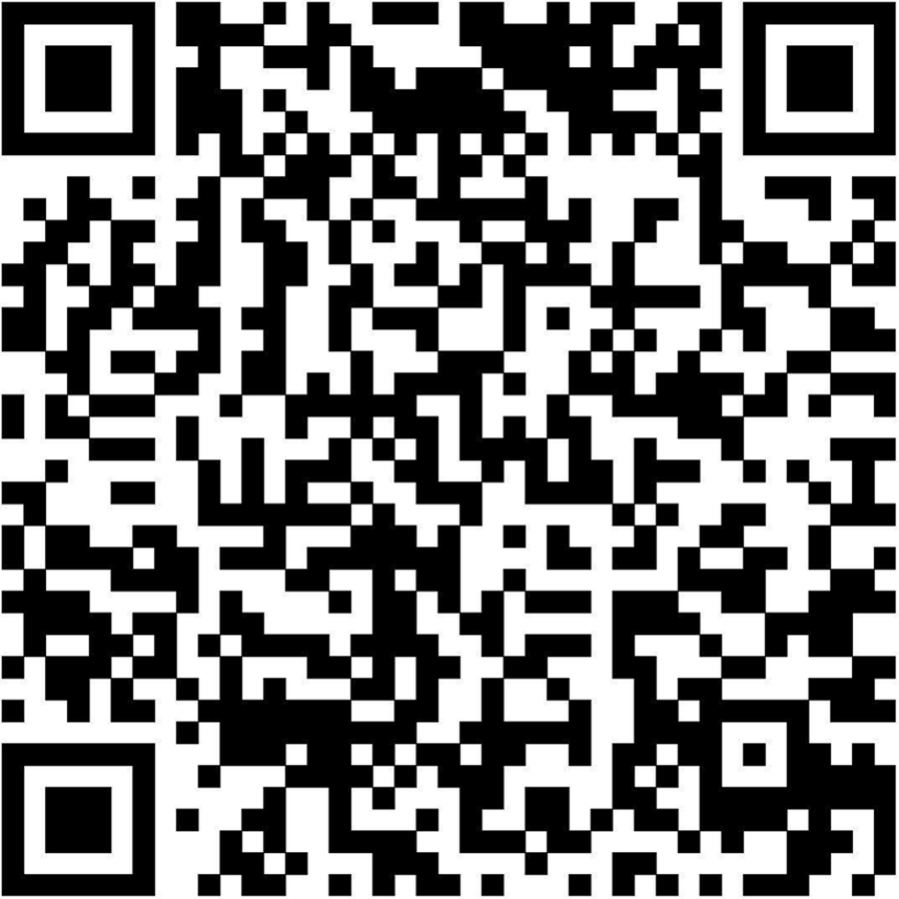


**Conclusion:** VASCA Magazine is a lighthouse project of cooperation and good partnership between Patient organizations’ representatives and clinicians of VASCERN-VASCA.

Due to its success, a new edition is being worked to be published early 2023.

## P43

### The European Joint Programme on Rare Diseases: an integrative approach fostering the RD research ecosystem

#### Juliane Halftermeyer^1,*^, Yanis Mimouni^2,*^, Daria Julkowska^2^

##### ^1^Inserm Transfert, Paris, France. ^2^Inserm, Paris, France

*Corresponding author: coordination@ejprarediseases.org

*Orphanet Journal of Rare Diseases* 2023: P43

The European Joint Programme on Rare Diseases (EJP RD) [1] aims to create an effective Rare Disease (RD) research ecosystem for progress, innovation benefiting patients.

Its governance allows alignment with the national strategies of the 35 countries, the European Commission and major players such as industry. Collaboration with the 24 European Reference Networks (ERNs) is strengthened.

The EJP RD financed (1) 52 multinational projects for € 67 million, including € 9.1 millions from EC through three Joint Transnational Calls focusing on acceleration of diagnosis, disease progression, RD mechanisms [2], effective therapies [3] and social sciences and humanities [4]; (2) three RD Research Challenges projects [5] focusing on public–private collaboration to develop therapeutic solutions; (3) 23 networking events supporting knowledge sharing [6]; (4) 3 demonstration [7] and 2 innovation [8] projects for RD clinical trials innovative methodologies; (5) 33 fellowships for young clinicians [9] and 20 transversal workshops for ERNs [10].

The Virtual Platform building blocks were developed including meta(data) models linking resources and considering access and reuse conditions. RD resources were made ready for linkage. Pilot tools querying resources to discover data are being tested. The beta version of the virtual platform discovery portal has been released [11]. 100 RD biological pathways are created and exploited through bioinformatic networks with multiomics data to accelerate RD diagnosis [12].

Sixteen training activities [13] and one MOOC [14] were delivered to nearly 500 stakeholders including EU 13 countries.

Multinational RD clinical trials and innovation management support services were implemented through European research infrastructures, supporting 16 clinical trials requests [15], 16 research projects for translational mentoring [16]. The Innovation Management Toolbox empowers researchers to conduct rigorous translational research has been released [17].

EJP RD is working on the sustainability of its activities and the generated results including the roadmap towards the future Rare Diseases Partnership [18].

**Acknowledgements**

The abstract is presented on behalf of the European Joint Programme on Rare Diseases—EJP RD. The EJP RD initiative has received funding from the European Union’s Horizon 2020 research and innovation programme under grant agreement N°825575.

**References**

1. https://www.ejprarediseases.org/
2. https://www.ejprarediseases.org/funded-projects-jtc2019/
3. https://www.ejprarediseases.org/funded-projects-jtc2020/
4. https://www.ejprarediseases.org/funded-projects-jtc2021/
5. https://www.ejprarediseases.org/fundings-and-calls/rare-diseases-challenges/
6. https://www.ejprarediseases.org/our-actions-and-services/funding-opportunities/funded-projects/networking-support-scheme-funded-networking-events/
7. https://www.ejprarediseases.org/funded-projects-demonstration/
8. https://www.ejprarediseases.org/funded-projects-internal-call-for-innovation-project-in-clinical-trials-methodology/
9. https://www.ejprarediseases.org/our-actions-and-services/funding-opportunities/funded-projects/ern-trainings-2/
10. https://www.ejprarediseases.org/our-actions-and-services/training-and-education/ern-workshops/
11. https://vp.ejprarediseases.org/discovery/
12. https://www.wikipathways.org/index.php/Portal:RareDisease
13. https://www.ejprarediseases.org/general-information-training/#
14. https://www.ejprarediseases.org/general-information-training/e-learning/
15. https://www.ejprarediseases.org/support-office/
16. https://www.ejprarediseases.org/mentoring/
17. https://imt.ejprarediseases.org/
18. https://ec.europa.eu/info/sites/default/files/research_and_innovation/funding/documents/ec_rtd_he-partnerships-rare-diseases.pdf

## P44

### RD-CODE PROJECT: Coding Rare Disease Diagnosis

#### Sylvie Maiella^1^, Deborah M. Lambert^2^, Virginia Corrochano^3^, Monica Mazzucato^4^, Francis Agius^5^, Clara Cavero-Carbonell^6^, Katerina Hanusova^7^, Monica Panzaru^8^, Cristina Rusu^8^, Oscar Zurriaga^6^, Miroslav Zvolsky^7^, Houda Ali^1^, Céline Angin^9^, Ines Hernando^10^, Kurt Kirch^11^, Valérie Lanneau^1^, Stefanie Weber^11^, Marc Hanauer^1^ and Ana Rath^1^

##### ^1^Inserm US14 – Orphanet, Paris, France. ^2^University College Dublin, Dublin, Ireland. ^3^CIBERER, Valencia, Spain. ^4^RD Coordinating Centre, Veneto region, Italy. ^5^Malta Mater Dei Hospital, Malta. ^6^Rare Diseases Research Unit, Foundation for the Promotion of Health and Biomedical Research in the Valencian Region, Valencia, Spain. ^7^Institute of Health Information and Statistics of the Czech Republic, Prague, Czech Republic. ^8^Grigore T Popa” University of Medicine and Pharmacy, Iasi, Romania. ^9^AP-HP – BNDMR, Paris, France. ^10^EURORDIS, Paris, France. ^11^BFARM, Koln, Germany

*Orphanet Journal of Rare Diseases 2023, supp P44*

Rare diseases (RD) are numerous and diverse, and require specialized management and care. Generic health information terminologies do not include all RD, and cannot identify them as rare. Their use in Health Information Systems results in inefficient health system planning and unmet RD patients’ needs. ORPHAcodes is the only terminology that specifically recognizes all RD.

The RD-CODE project, co-funded by the European Union’s Third Health Programme and coordinated by INSERM, supported Member States in implementing ORPHAcodes into routine codification systems and improved RD data-gathering in a standardised and consistent way. In particular, the project has:

1. Supported the implementation of ORPHAcodes in Czech Republic, Malta, Romania and Spain.
2. Identified the needs in terms of resources for effective ORPHAcodes implementation in different settings.
3. Refined and expanded the available procedures and guidelines for coding (including for undiagnosed diseases) to guarantee the appropriate use of the resources allowing comparability across countries and settings.
4. Developed assistance and tooling to improve the codification experience and accuracy.

To assess if ORPHAcodes data can be combined across data sources and countries, a preliminary study looked at aggregated data collected in the Czech Republic, Malta, Romania, Spain along with data from the Veneto region of Italy. No individual patient data was used. The number and type of ORPHAcodes and rare diseases were counted and compared. 3,137 ORPHAcoded from 5 countries were included. This preliminary study has shown that:

- Comparability increases when ORPHAcodes are used at population level.
- ORPHAcodes capture effectively ultra-rare diseases, which are not captured by the widely used ICD-10.
- ORPHAcodes and coding guidelines knowledge is essential to ensure correct code assignation.
- Historization of codes according to the annual releases of the Nomenclature pack is essential.

Overall this shows that ORPHAcodes are a versatile coding resource which can be effectively introduced in different settings preserving consistency. The project has identified « do and don’ts» to guide the implementation process and also has developed many resources, both for general public use and technical guides available for the community at large on the www.rd-code.eu website.

Finally, the project participants have set up a Community of Practice to capitalise on the project’s legacy (Community.Of.Practice@orphacodes.org), to serve as a collaborative environment to move forward in the process of RD coding to increase patients’ visibility, including undiagnosed ones, across a diversity of settings.

## S1

### Inclusive educational tools for teachers with students with rare diseases: the CREDIBLE project

#### Oscar R. Lozano^1^

##### ^1^Dpt. Didàctica de les Ciències. Universitat de València. Valencia, Spain

*Orphanet Journal of Rare Diseases 2023, supp S1*

Under the umbrella of the Erasmus + program, 10 organizations (educational centres, national federations of patients with RD, government entities, companies and universities from 5 different countries, Romania, Latvia, Greece, the United Kingdom and Spain), are associated to generate useful tools for improve the educational inclusion of children with rare diseases. Based on the demands of the Spanish associations of people with rare diseases about the shortcomings of the educational system [1, 2] and the experiences of the members of the consortium, three Intellectual Outputs have been developed to help teaching work in the effective development of inclusive education. Some of these demands included incorporation of the children to conventional schools (if they are properly adapted) and an adequate teacher training. Collaborative work creating professional networks, and adapting results from the research on the field in practical and helpful documents, are the pillars of the tools generated by the consortium which are:

1. *Platform for the exchange of experience*s. A platform has been created in which teachers who have had a case of children with rare diseases in their classrooms can share this experience and teachers who are facing this challenge for the first time can search for previous experiences of their classmates from anywhere in the world. world. world. world.
2. *Teacher training course*. A self-training course has been developed, freely available on several European educational resource websites, offering strategies and methodologies drawn from educational research for effective inclusive education of children with rare diseases.
3. *Pedagogic guide*. A manual-guide has been prepared that combines health and pedagogical aspects of the most prevalent rare diseases and with the greatest expectation of success in the educational inclusion of children with rare diseases.

All results are free and available at different online sources [3, 4].

**References**

1. FEDER (2013). *Informe de educación en enfermedades raras*. Retrieved 20/07/22 from https://obser.enfermedades-raras.org/wp-content/uploads/2019/01/Informe-de-Educaci%C3%B3n_2014.pdf
2. Gaintza Jauregi, Z., Aróstegui Barandica, I., Berasategi Sancho, N., Ozerinjauregi Beldarrain, N., Darretxe Urrutxi, L., Orcasitas García, J. R., & Monzón González, F. J. (2015). *La innovación escolar desde la perspectiva de personas con enfermedades raras en el País Vasco: historias de vida, prácticas escolares, necesidades del sistema educativo y propuestas de mejora para una escuela y sociedad inclusiva.* Retrieved 20/07/22 from https://addi.ehu.es/bitstream/handle/10810/18420/La%20Innovacion%20Escolar_libro.pdf?sequence=1&isAllowed=y
3. European commission (n.d). https://erasmus-plus.ec.europa.eu/projects/search/details/2019-1-ES01-KA201-063925
4. CREDIBLE (n.d.). https://erasmuscredible.eu/

## S2

### Evaluating the cost of developing, manufacturing and commercialising gene therapies and implications for commercial attractiveness

#### Tim Wilsdon^1^, Artes Haderi^1^, Anna Robson^1^, Nicolas Koebel^2^, Francis Pang^2^

##### ^1^Charles River Associates, London, UK. ^2^Orchard Therapeutics, London, UK

*Corresponding author: francis.pang@orchard-tx.com

*Orphanet Journal of Rare Diseases* 2023: S2

**Background:** As more gene therapies become available, there is increasing debate around their price levels and affordability and the potential returns to manufacturers. This study describes the cost of developing, manufacturing and supplying gene therapies and assesses the implications for financial viability.

**Materials and methods:** A three-step approach was adopted to identify and quantify the key specificities for the development, manufacturing and commercialisation of gene therapies, with an emphasis on the unique aspects of the ex vivo approach: (i) A literature review (2016–2020) was conducted to set out the value chain for gene therapies, which was validated through interviews with functional experts possessing real-world operational experience within an ex vivo gene therapy company; (ii) Differences from conventional treatment modalities were validated with 11 external experts including researchers, gene therapy firms, investors, non-governmental organisations and contract research organisations; (iii) Public data were collected to quantify the cost associated with each key ex vivo gene therapy specificity identified. Finally, a financial model informed by these insights was developed as a framework for quantifying costs and to evaluate financial viability.

**Results:** The total industry out-of-pocket cost per approved gene therapy is estimated to be $1.98bn and the total capitalised cost per approved gene therapy to be $3.38bn. Using industry assumptions, financial returns were modelled producing an “upside” illustrative internal rate of return (IRR) of 5.6%, which is slightly below cost of capital estimates for pharmaceutical developers, and a “downside” IRR of 0.3%.

**Conclusions:** This analysis suggests that the development costs of an approved gene therapy are higher than for a conventional medicine and it is more challenging to achieve commercial viability. The increased complexity and uncertain prospects of financial returns for gene therapy developers indicate that urgent action is needed within the healthcare ecosystem to sustain the development and availability of these transformational medicines.

