# Correction: Meeting abstracts from the 11th edition of the European conference on Rare Diseases & Orphan Products (ECRD) 2022

**DOI:** 10.1186/s13023-023-02960-7

**Published:** 2023-11-30

**Correction: Orphanet Journal of Rare Diseases (2023) 18:118** **https://doi.org/10.1186/s13023-023-02707-4**

**P3**

**Innovating for people living with a rare disease: Why partnerships are key for the European OMP ecosystem**

Christian Jervelund^1,*^, Julia S. Wahl^2^, Tuomas Haanperä^3^, Emil Löfroth^4^, Nikolaj Siersbæk^1^, Elisa Pau^2^, Laura Virtanen^1^, Toon Digneffe^5^ and Luana Banu^5^

^1^Copenhagen Economics, Copenhagen, Denmark

^2^Copenhagen Economics, Brussels, Belgium

^3^Copenhagen Economics, Helsinki, Finland

^4^Copenhagen Economics, Stockholm, Sweden

^5^Takeda Pharmaceutical Company, Opfikon, Switzerland

*Corresponding author: cj@copenhageneconomics.com

**Orphanet Journal of Rare Diseases** **2023:** **P3**

Following publication of the original article [1], we have been notified that Fig. 1 in abstract **“Innovating for people living with a rare disease: Why partnerships are key for the European OMP ecosystem” was published incorrectly.** Please see below incorrect Fig. 1:

**Figure:**
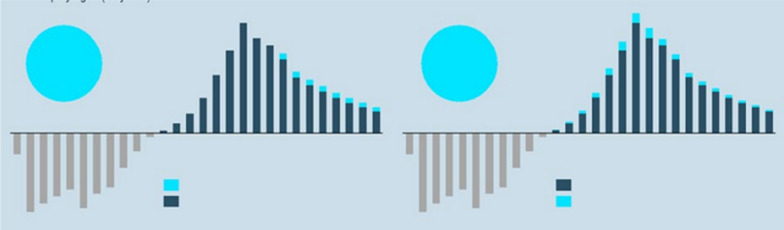
**Fig. 1** Effect of an increase in length of ME (left) and partnership solution (right) on the rNPV of a hypothetical ultra-rare OMP [3]. ME = market exclusivity, rNPV = risk-adjusted net present value

Correct Fig. 1 is:

**Figure:**
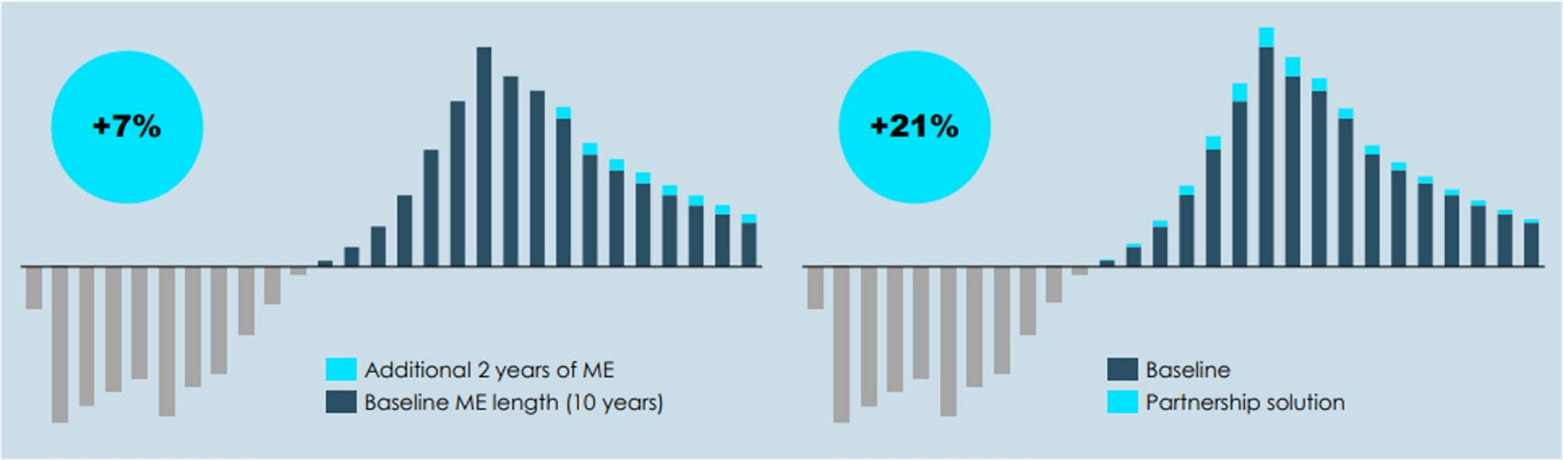
**Fig. 1** Effect of an increase in length of ME (left) and partnership solution (right) on the rNPV of a hypothetical ultra-rare OMP [3]. ME = market exclusivity, rNPV = risk-adjusted net present value

The original article was updated.
